# Neuronal PPP2R5C in plasma is a potential biomarker for early diagnosis of Alzheimer’s disease

**DOI:** 10.1016/j.xcrm.2026.102631

**Published:** 2026-02-19

**Authors:** Shilin Luo, Hui Liu, Tingting Xiao, Yunni Li, Xixi Liu, Xuewen Xiao, Xinxin Liao, Yingzi Liu, Yafang Zhou, Jun-Ling Wang, Jifeng Guo, Tian Tu, Xiaoxin Yan, Beisha Tang, Zhentao Zhang, Bin Jiao, Lu Shen

**Affiliations:** 1 Department of Neurology, Xiangya Hospital, Central South University, Changsha, Hunan 410008, China; 2 National Clinical Research Center for Geriatric Diseases (Xiangya Hospital), Changsha, Hunan 410008, China; 3 Engineering Research Center of Hunan Province in Cognitive Impairment Disorders, Changsha, Hunan 410008, China; 4 Hunan International Scientific and Technological Cooperation Base of Neurodegenerative and Neurogenetic Diseases, Changsha, Hunan 410008, China; 5 Department of Neurology, Renmin Hospital of Wuhan University, Wuhan 430060, China; 6 Department of Geriatric Neurology, Xiangya Hospital, Central South University, Changsha, Hunan 410008, China; 7 Department of Anatomy and Neurobiology, Xiangya Medical School, Central South University, Changsha, Hunan 410013, China; 8 Furong Laboratory, Central South University, Changsha, Hunan 410008, China

**Keywords:** Alzheimer’s disease, biomarker, PPP2R5C, Tau, autophagy

## Abstract

Early intervention is the most effective strategy to impede the progression of Alzheimer’s disease (AD), depending on the identification of early diagnostic biomarkers. Here, we isolate neuron-derived exosomes (NDEs) from plasma of familial AD (FAD), presymptomatic FAD (pre-FAD), and healthy controls (cognitively normal [CN]), followed by label-free liquid chromatography-tandem mass spectrometry (LC-MS/MS) analysis. A specific peptide from protein phosphatase 2 regulatory subunit B'β (PPP2R5C) shows a progressive decrease from CN to pre-FAD and FAD patients. This decline is further validated in plasma NDEs and brain tissue from amnestic mild cognitive impairment (aMCI) and sporadic AD (SAD) patients. Two independent cohorts confirm the early and differential diagnostic value of plasma PPP2R5C. Immunohistochemistry of Tau Braak-staged brains reveals PPP2R5C reduction preceding Tau hyperphosphorylation. Mechanistically, PPP2R5C interacts with Tau, reducing Tau levels and phosphorylation via unc-51-like kinase 1 (ULK1)-dependent autophagolysosomal activation and PP2A regulation. Our findings suggest that plasma PPP2R5C has the potential to serve as an ideal biomarker for the early diagnosis of AD.

## Highlights

• PPP2R5C is an early-declining marker across pre-symptomatic, familial, and sporadic AD
• Multi-cohort demonstrates early and differential diagnostic potential of PPP2R5C for AD
• PPP2R5C reduction precedes tau hyperphosphorylation
• PPP2R5C reduces Tau and p-Tau via ULK1-dependent autophagolysosomal and PP2A activation

## Graphical abstract

**Figure:**
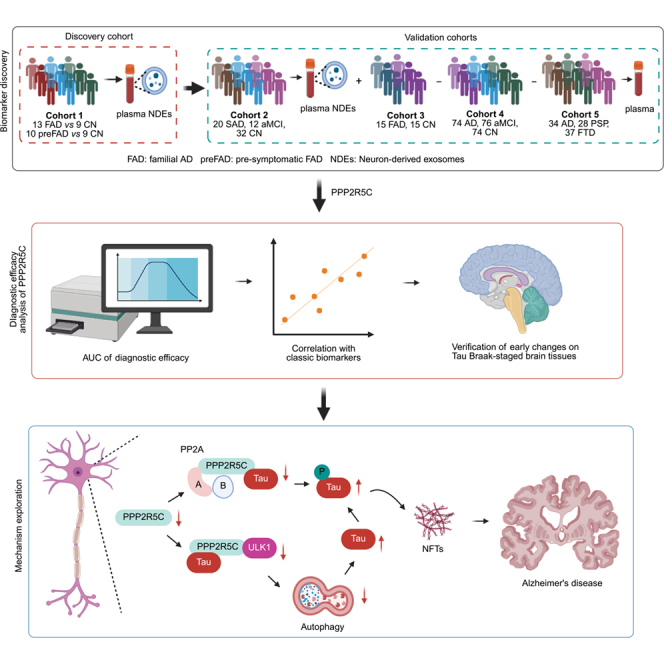

## Teaser

Luo et al. identify PPP2R5C in plasma as an early biomarker of Alzheimer’s disease that declines prior to tau hyperphosphorylation. PPP2R5C modulates tau homeostasis via PP2A activation and ULK1-dependent autophagolysosomal pathways, enabling early diagnosis and providing mechanistic insight into AD pathogenesis.

## Introduction

Alzheimer’s disease (AD), the predominant kind of dementia, substantially impacts the aging population worldwide, presenting a profound healthcare challenge.^1^ AD is characterized by a gradual deterioration in memory and cognitive abilities. Pathologically, it is identified by two primary features: extracellular senile plaque composed of aggregated amyloid beta (Aβ) and intracellular neurofibrillary tangles (NFTs) consisting of hyperphosphorylated Tau.^2^ Overwhelming evidence has confirmed that patients suffering from AD were experiencing pathological alterations and brain damage decades before the onset of symptoms.^3^^,^^4^^,^^5^ As a result, any disease-modifying treatment, particularly the clinical application of Aβ monoclonal antibody drugs, will only be most effective if it begins in the early stages of AD, which is dependent on early warning and diagnosis.

Current AD diagnostic techniques, such as cerebrospinal fluid (CSF) analysis and positron emission tomography, are inconvenient and costly, restricting widespread adoption in clinical practice.^6^ Therefore, scientists are switching AD diagnosis from the brain signals to peripheral biomarkers and have shifted the treatment from the late stages of the disease toward the earlier stages, introducing the possibility of pre-symptomatic diagnosis,^7^ especially for developing blood-based markers for AD early diagnosis has receiving considerable attention,^8^^,^^9^^,^^10^ which is further reflected in p-Tau217 assay in plasma was recommended in the revised criteria for diagnosis of AD.^11^^,^^12^ However, biomarkers based on peripheral blood are still being explored due to the complex pathogenesis of AD, such as the “hub proteins” representing the AD plasma protein profile^13^^,^^14^ and eight serum protein panels for early AD diagnosis.^15^ Thus, it holds significant potential to identify biomarkers for the early diagnosis of AD based on blood samples in clinical cohorts and to elucidate the underlying mechanism thoroughly.

Hyperphosphorylation of Tau disrupts the stability of cellular microtubules, resulting in the formation of NFTs, which causes neuronal dysfunction and death.^16^ Given the crucial role of NFTs in the progression of AD, Tau levels at many different phosphorylation sites (p-tau181, p-tau217, and p-tau231) can be utilized as potential biomarkers for diagnosing AD. Protein phosphatase 2A (PP2A) is an important serine/threonine phosphatase that accounts for approximately 70% of Tau phosphatase activity in the human brain.^17^ It performs as a heterotrimeric protein complex consisting of a scaffold subunit (or A subunit), a catalytic subunit (or C subunit), and its core element associated with variable regulatory subunits (B subunit).^18^ Among them, the protein phosphatase 2 regulatory subunit B'β (PPP2R5C or B56) is essential for regulating cell growth, specialization, and conversion by promoting the removal of phosphate groups from distinct sites on the p53 protein.^19^ Meanwhile, PPP2R5C was highly expressed in human brain tissue, and a single-nucleotide polymorphism in the *PPP2R5C* gene was reported to have a significant association with the risk of late-onset AD.^20^ However, the mechanism of PPP2R5C on Tau remains unknown, and it is unclear whether it can act as a biomarker for the early diagnosis of AD.

In this study, PPP2R5C levels in plasma at different stages of AD were analyzed, and its efficacy was compared with other well-studied AD biomarkers due to the discovery that PPP2R5C in neuron-derived exosomes (NDEs) decreased significantly as AD progressed. In addition, as the core regulatory subunit of PP2A, the mechanism by which PPP2R5C regulates both total Tau and phosphorylated Tau has been elucidated, demonstrating its potential as an early diagnostic biomarker for AD.

## Results

### PPP2R5C levels in NDEs are decreased during AD progression

**Figure 1:**
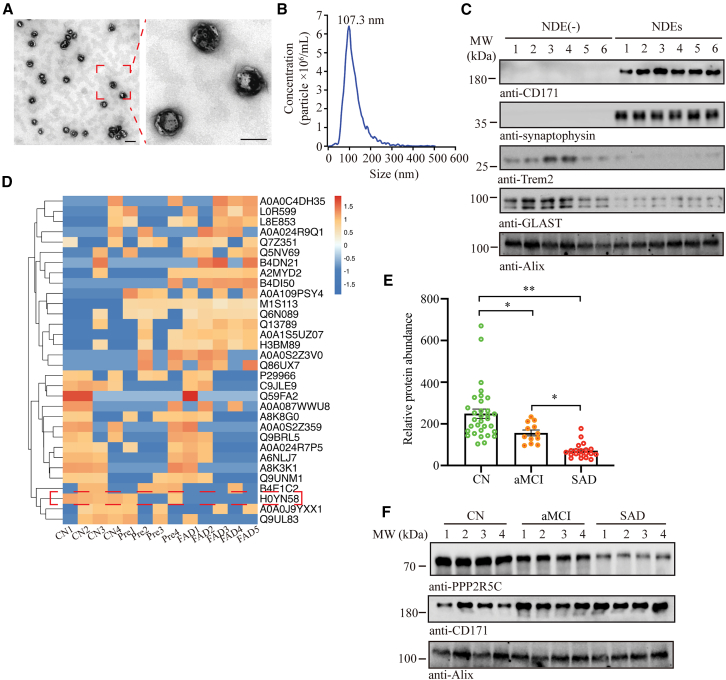
Alterations in PPP2R5C protein levels in NDEs from the plasma of AD patients (A) Representative TEM image of NDEs. Scale bars: 1 μm for low magnification and 100 nm for high magnification. (B) Nanoparticle tracking analysis of NDEs, confirming the expected size range. (C) Western blot analysis validation of NDEs and non-NDEs, using neuronal exosome markers, CD171 and synaptophysin; a microglia-derived exosome marker, Trem2; an astroglia-derived exosome marker, GLAST; and the exosome marker, Alix. (D) Label-free proteomic analysis of plasma NDEs showing differences in PPP2R5C protein levels among CN controls (*n* = 4), pre-symptomatic familial AD mutation carriers (pre-FAD) (*n* = 4), and familial AD (FAD) patients (*n* = 5). (E) Targeted protein analysis comparing PPP2R5C protein levels in plasma NDEs across AD (*n* = 20), aMCI (*n* = 12), and CN groups (*n* = 32). (F) Western blot analysis showed the difference in PPP2R5C levels in NDEs between AD, aMCI, and CN groups. All the western blot data are representative of three independent experiments. Quantification data are expressed as mean ± SEM (∗*p* < 0.05 and ∗∗*p* < 0.01 with Student’s *t* test).

To explore the specific early diagnostic markers of AD in neurons, we isolated NDEs from plasma in cohort 1. Transmission electronic microscopy (TEM) images showed the morphology of NDEs (Figure 1A), and the particle size of TEM matched with the nanoparticle tracking analysis, displaying the size of NDEs to be distributed mainly between 70 and 150 nm (Figure 1B). The source of NDEs was validated using a marker combination composed of neuronal CD171 and synaptophysin,^21^ Trem2 (a microglia-derived exosomes marker),^22^ GLAST (astroglia-derived exosomes marker),^23^ and exosomal Alix in immunoblotting analysis (Figure 1C). Further, we conducted proteomic analysis by liquid chromatography-tandem mass spectrometry (LC-MS/MS). The results demonstrated that a peptide named H0YN58, specific to PPP2R5C, gradually decreased significantly in healthy controls, pre-familial AD (FAD), and FAD patients from cohort 1 (Figure 1D). To substantiate this finding, we identified the differential abundances of PPP2R5C in NDEs from cohort 2, including sporadic AD, individuals with amnestic mild cognitive impairment (aMCI), and healthy controls (cognitively normal [CN]), using parallel reaction monitoring (PRM)-based targeted proteomics analysis. In alignment with the result from LC-MS/MS detection in cohort 1, PPP2R5C levels were significantly decreased in both aMCI and AD compared with those in the CN group, and the degree of decline was significantly higher in AD than in the aMCI group (Figure 1E). Finally, we performed PPP2R5C immunoblot detection on randomly selected NDE samples, and the results were still consistent with the downward trend (Figure 1F). Thus, these findings indicate that neuron-derived PPP2R5C has potential as biomarker for early AD diagnosis.

### Plasma PPP2R5C has significant early diagnostic and differential diagnostic efficacy for AD

**Figure 2:**
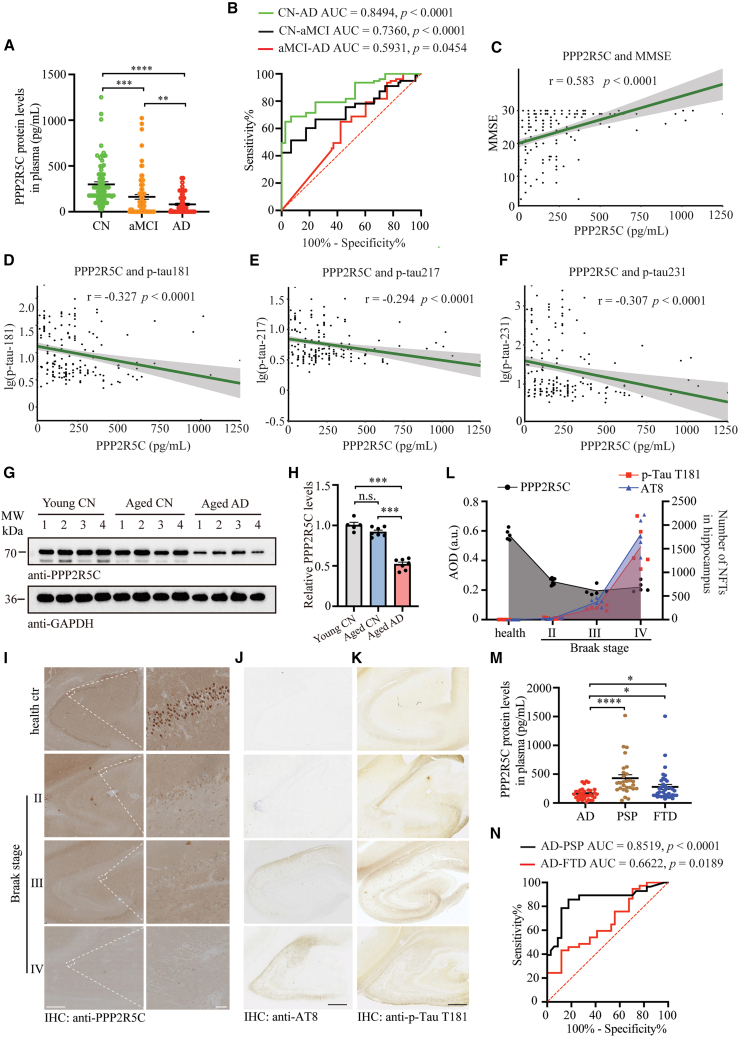
Diagnostic utility of plasma PPP2R5C protein levels in AD (A) Plasma PPP2R5C protein levels in AD, aMCI, and CN groups by ELISA technology. AD, *n* = 74. aMCI, *n* = 76. CN, *n* = 74. (B) Evaluation of the diagnostic performance of plasma PPP2R5C protein levels in distinguishing AD from aMCI and CN. (C–F) Spearman correlation analysis between plasma PPP2R5C levels, Mini-Mental State Examination (MMSE) scores, and other AD biomarkers, including p-Tau T181, p-Tau S231, and p-Tau T217. (G) Representative western blots for PPP2R5C in brain tissues from young individuals, cognitively normal elderly individuals, and AD patients. (H) Quantitation of PPP2R5C levels, normalized to young CN (*n* = 5 or 7). (I–K) Representative immunohistochemistry images of PPP2R5C (I), AT-8 (J), and p-Tau T181 (K) levels in the hippocampus of healthy controls and Braak-graded AD brain slices. Scale bars: 1 mm in (I, left), 50 μm in (I, right), and 1 mm in (J and K). (L) Quantitation of PPP2R5C, p-Tau T181, and AT8 levels (sample quantify, health ctr: 5, Braak II: 9, Braak III: 5, and Braak IV: 4). (M) Difference of plasma PPP2R5C protein levels among AD (*n* = 34), PSP (*n* = 28), and FTD (*n* = 37) patients using ELISA. (N) ROC curve analysis assessing the discriminative power of plasma PPP2R5C protein levels for distinguishing AD from PSP and FTD. All the western blot data are representative of three independent experiments. Quantification data are expressed as mean ± SEM (∗*p* < 0.05, ∗∗*p* < 0.01, ∗∗∗*p* < 0.001, and n.s., no statistics with one-way ANOVA with Tukey’s multiple comparisons test).

Given the challenges associated with the cost and the substantial sample volumes required for isolating NDEs from plasma, we explored PPP2R5C in peripheral blood plasma as a potential biomarker for early AD diagnosis. The flowchart for the discovery and validation process is shown in Figure S2. First, we validated the plasma PPP2R5C concentration in a separate FAD cohort (cohort 3) from the Cognitive Impairment Multicenter Database and Collaborative Network in China (CI-MDCNC) using an ELISA assay. Convincingly, PPP2R5C was significantly decreased in FAD patients (Figure S3A). Second, when expanding PPP2R5C to sporadic AD patients, the mean levels of plasma PPP2R5C in the aMCI and AD groups, compared with healthy controls, were significantly reduced by approximately 61.3% (*p* = 0.0074) and 31.6% (*p* < 0.0001), respectively. Compared with the mean level in the aMCI group, the mean level of plasma PPP2R5C in the AD was reduced by approximately 52.1% (*p* = 0.0072) (inter-assay = 5.2) (Figure 2A). The changing trend of PPP2R5C levels in plasma was exactly the same as that in NDEs, indicating the feasibility of directly detecting PPP2R5C in plasma. Then, the early diagnostic and differential diagnostic ability of PPP2R5C in plasma for AD was analyzed. Diagnostic accuracy was evaluated using receiver operating characteristic (ROC) curve analysis. Initially, plasma PPP2R5C demonstrated significant diagnostic ability for AD (area under the curve [AUC] = 0.8494, *p* < 0.0001) and for aMCI (AUC = 0.7360, *p* < 0.0001) (Figure 2B). Plasma PPP2R5C also showed a certain degree of differential diagnosis of aMCI from AD (AUC = 0.5931, *p* = 0.0454) (Figure 2B). Notably, combining PPP2R5C levels with age and gender yields an excellent diagnostic value, with an AUC of 0.74 (Figure S3B). To further investigate PPP2R5C alterations in the blood, plasma samples from cohort 3 were subsequently tested for PPP2R5C and other biomarkers. A series of correlation analyses were performed, and the results showed a significant positive correlation between plasma PPP2R5C levels and MMSE (*r* = 0.538, *p* < 0.0001) (Figure 2C). Plasma PPP2R5C significantly negatively correlated with plasma p-tau181 (*r* = −0.327, *p* < 0.0001), plasma p-tau217 (*r* = −0.294, *p* < 0.0001), and plasma p-tau231 (*r* = −0.307, *p* < 0.0001) (Figures 2D–2F). However, plasma PPP2R5C demonstrates no significant correlations with neurofilament high chain (NFL) (*r* = 0.159, *p* = 0.0171), Aβ42 (*r* = −0.108, *p* = 0.1062), and Aβ40 (*r* = 0.0849, *p* = 0.2047) (Figures S3C–S3E). Further, given that p-tau217 is considered the most promising blood biomarker for AD diagnosis,^24^ we performed a combined diagnostic analysis of PPP2R5C and p-tau217, along with other conventional variables, which demonstrated that PPP2R5C offers meaningful incremental diagnostic utility, especially in distinguishing between CN and MCI. The AUC, sensitivity, specificity, and accuracy for all analysis models are listed in Table S2. Meanwhile, we detected a significant decrease in PPP2R5C in aged AD but not in aged healthy brains compared to young healthy brains (Figures 2G and 2H). Furthermore, to investigate which of PPP2R5C diminishment or NFT accumulation will occur first in the progression of AD, we performed IHC staining using a series of healthy controls and Braak-graded AD brain slices with specific PPP2R5C and p-Tau antibodies (Figures 2I–2K). The results showed that although intraneuronal PPP2R5C expression in the hippocampus of the Braak II brain slice was dramatically diminished, NFTs were still barely present, as indicated by AT8 and p-Tau T181, which demonstrated sporadic immune responses. In Braak II and IV levels where NFTs were heavily accumulated, PPP2R5C was in a stable state of low expression (Figure 2L). Finally, to explore the differential diagnostic efficacy of PPP2R5C, plasma PPP2R5C levels in the AD, progressive supranuclear palsy (PSP), and frontotemporal dementia (FTD) groups were compared, revealing that the average concentration of PPP2R5C was significantly lower in the AD group than in the other two groups (Figure 2M), which demonstrated high diagnostic efficacy in the differential diagnosis of AD from PSP (AUC = 0.8519, *p* < 0.0001) and moderate efficacy in AD from FTD (AUC = 0.6622, *p* = 0.0189) (Figure 2N). Thus, the above results revealed that plasma PPP2R5C has the potential for early diagnostic and differential diagnostic efficacy for AD.

### PPP2R5C associates with Tau and attenuates its expression

**Figure 3:**
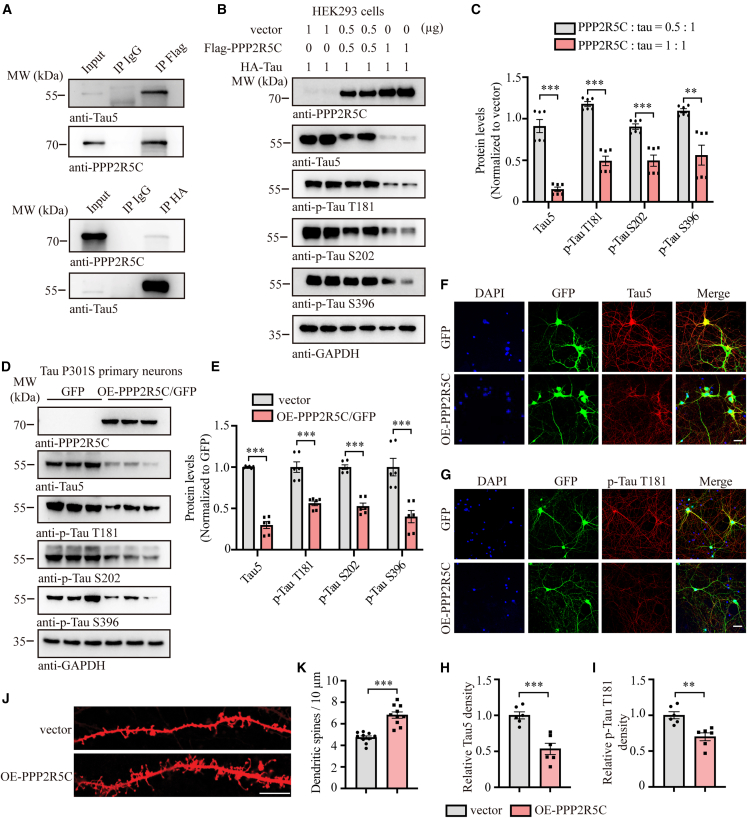
PPP2R5C interacts with Tau and attenuates its expression (A) Co-immunoprecipitation analysis demonstrating the interaction between PPP2R5C and Tau protein. (B) Representative western blots immunostained for t-Tau, p-Tau T181, p-Tau S202, and p-Tau S396, in HEK293 cell lysates following PPP2R5C overexpression. (C) Quantification of t-Tau, p-Tau T181, p-Tau S202, and p-Tau S396, normalized to vector (*n* = 6). (D) Representative western blots showing t-tau, p-Tau T181, p-Tau S202, and p-Tau S396 in primary neurons from Tau P301S mice following PPP2R5C overexpression. (E) Quantification of t-Tau, p-Tau T181, p-Tau S202, and p-Tau S396 levels, normalized to vector (*n* = 6). (F and G) Double-labeling immunofluorescence analysis of GFP (green) and t-Tau/p-Tau T181 (red) was conducted on primary Tau P301S neurons after PPP2R5C overexpression. Scale bars, 10 μm. (H and I) Quantification of t-tau and p-Tau T181 fluorescence intensities (*n* = 6). (J) Representative DiI staining shows the difference in spine density in tau P301S neurons after PPP2R5C overexpression. Scale bar: 10 μm. (K) Quantification of the density of spines (*n* = 10). All the western blot data are representative of three independent experiments. Quantification data are expressed as mean ± SEM (∗∗*p* < 0.01 and ∗∗∗*p* < 0.001 with Student’s *t* test).

PPP2R5C acts as one of the regulatory subunits of PP2A and shows a gradual decline during AD progression. Thus, we attempted to find the underlying mechanisms between PPP2R5C and Tau. To examine whether PPP2R5C associates with Tau, we first performed FLAG and hemagglutinin (HA) pull-down assays from cells co-transfected with both FLAG-PPP2R5C and HA-tagged Tau constructs. Interestingly, PPP2R5C displayed a strong interaction with Tau (Figure 3A). To further explore whether PPP2R5C dictates Tau protein stability, we transfected different amounts of FLAG-PPP2R5C constructs into HEK293 cells with equal HA-Tau and found that both total Tau (t-Tau) and phosphorylated Tau (p-Tau) levels were gradually diminished when PPP2R5C was increased (Figures 3B and 3C), which was confirmed in endogenous Tau after overexpressing PPP2R5C in Tau P301S primary neurons (Figures 3D and 3E). Moreover, immunofluorescence (IF) staining demonstrated that t-Tau and p-Tau181 in Tau P301S primary neurons were significantly attenuated after they were infected with GPF-PPP2R5C lentivirus (Figures 3F–3I), followed by an increase in dendrite spine density in Dil staining (Figures 3J and 3K). Contrary to overexpression studies, we employed a targeted approach to silence PPP2R5C expression in Tau P301S primary neurons using sh-GFP-PPP2R5C lentivirus. As expected, both immunoblotting and IF staining demonstrated that t-Tau and p-Tau were elevated (Figures S4A–S4F), and the dendritic spine density was significantly decreased after virus infection (Figures S4G and S4H). Therefore, PPP2R5C interacts with Tau and attenuates its expression both in total and phosphorylated levels.

### Overexpression of PPP2R5C decreases Tau and AD-like pathogenesis in Tau P301S mice

Inspired by our initial research that PPP2R5C was discovered in NDEs, we characterized the expression pattern of PPP2R5C in major nerve cells in wild-type (WT) mice brains by performing co-IF staining using specific anti-PPP2R5C with NeuN (neuron marker), GFAP (astrocyte marker), and Iba1 (microglia marker). The results demonstrated that PPP2R5C localizes in NeuN-positive cells but was not co-expressed with either GFAP or Iba1, indicating that PPP2R5C is predominantly expressed in neurons (Figure S5A). Additionally, both immunoblotting and IHC staining showed that PPP2R5C was significantly decreased in Tau P301S mice compared to their age- and sex-matched WT littermates (Figures S5B–S5E).

**Figure 4:**
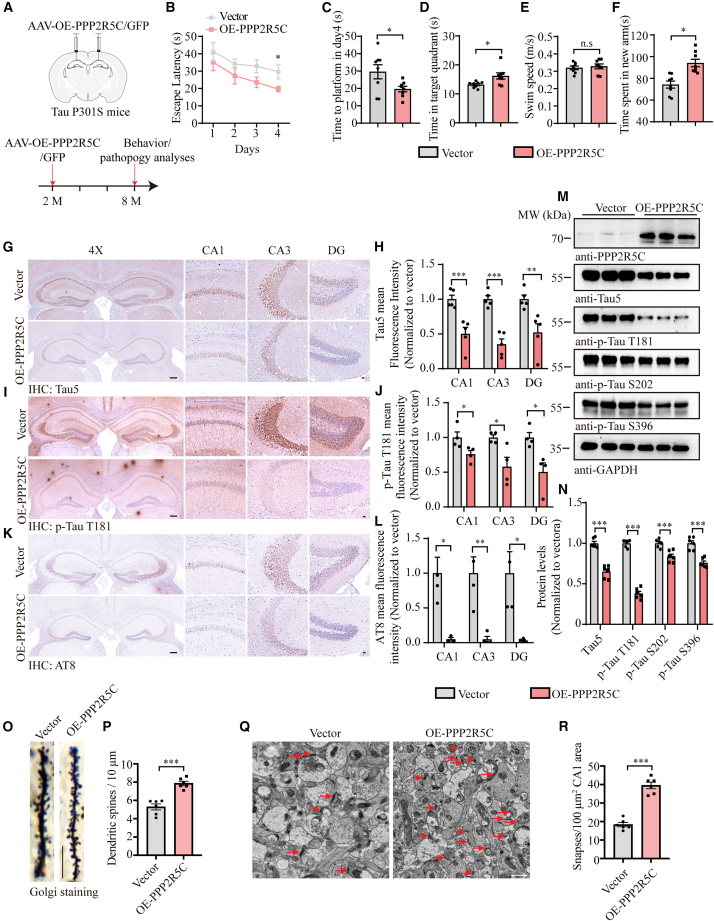
Overexpression of PPP2R5C in Tau P301S mice reduces AD-like pathogenesis and rescues cognitive function (A) Schematic representation of the experimental design. Two-month-old Tau P301S mice were injected with either AAV-hSyn-EGFP or AAV-hSyn-PPP2R5C-EGFP. Mice were sacrificed 6 months after AAV injection. (B and C) Morris water maze analysis as escape latency (s) and escape latency on day 4 (s). (D) Probe trial performance of Morris water maze test. (E) Swim speed of mice injected with AAVs encoding EGFP or PP2R5C-EGFP (*n* = 8–10 mice per group). (F) Time spent in the novel arm in the Y-maze test (*n* = 8–10 mice per group). (G, I, and K) Representative immunostaining images of t-Tau, p-Tau T181, and AT8 in the hippocampus of Tau P301S mice injected with AAVs encoding EGFP or PPP2R5C-EGFP. Scale bars: 200 μm for 4× images and 20 μm for magnified images. (H, J, and L) Quantification of immunoreactivity for t-Tau, p-Tau T181, and AT8 (*n* = 5 mice per group). (M and N) Representative western blots showing Tau pathology in mouse brain tissue following PPP2R5C overexpression (*n* = 6 mice per group). (O) Golgi staining revealed the dendritic spines in the apical dendritic layer of the CA1 region. Scale bar, 10 μm. (P) Quantification of spine density (*n* = 6 mice per group). (Q) Representative electron microscopy of the synapse structures. Arrows indicate synapses. Scale bar, 1 μm. (R) Quantification of synaptic density (*n* = 6 mice per group). All the western blot data are representative of three independent experiments. Quantification data are expressed as mean ± SEM (∗*p* < 0.05, ∗∗*p* < 0.01, ∗∗∗*p* < 0.001, and n.s., no statistics, Student’s *t* test).

To assess whether overexpression of PPP2R5C can reverse the cognitive deficits, we injected adeno-associated viruses (AAVs) encoding either GFP or human PPP2R5C with a GFP tag and human synapsin 1 gene promoter into the hippocampus of 2-month-old Tau P301S mice. After 6 months of infection, we assessed spatial learning and memory using the Morris water maze and Y-maze tests (Figure 4A). During the four acquisition days, mice with PPP2R5C overexpression exhibited a gradually decreased latency to reach the platform when compared with the vehicle-treated mice, indicating PPP2R5C averted the deterioration of spatial learning ability of Tau P301S mice (Figures 4B and 4C). In the probe trial, PPP2R5C-overexpressed Tau P301S mice spent more time in the target quadrant, even though the swimming speed did not alter (Figures 4D and 4E). Complementary to the Morris water maze findings, the Y-maze test demonstrated that mice with PPP2R5C overexpression spent significantly more time exploring the novel arm, as opposed to their control counterparts, signifying a potential enhancement in cognitive function (Figure 4F). In addition, pathological examination was performed after the behavioral tests. Tau5, p-Tau T181, and AT8 staining showed that AAV-PPP2R5C attenuated Tau pathology and Tau phosphorylation to a greater extent than that seen with control virus-treated 5xFAD mice (Figures 4G–4L), which was further confirmed in the IF staining with the same antibodies (Figures S6A–S6F) and the immunoblotting assay (Figures 4M and 4N). Neuronal loss and the activation of microglia and astroglia were diminished after the PPP2R5C overexpression (Figures S6G–S6J). Finally, to investigate the effect of PPP2R5C on synaptic loss, we first assessed the density of dendritic spines along individual dendrites of pyramidal neurons by Golgi staining. The results showed that the spine density was noticeably restored in PPP2R5C-treated Tau P301S mice compared with the vehicle group (Figures 4O and 4P). Since one dendritic spine can form more than one synapse, we further quantified the densities of synapses in the CA1 by electron microscopy (EM). As expected, the synaptic density in PPP2R5C-treated mice was higher than that in vector-treated ones (Figures 4Q and 4R). Together, these results support that PPP2R5C prevents synaptic loss in Tau P30S mice by attenuating Tau pathology.

### Blocking of PPP2R5C accelerates Tau pathology and worsens cognitive dysfunctions

**Figure 5:**
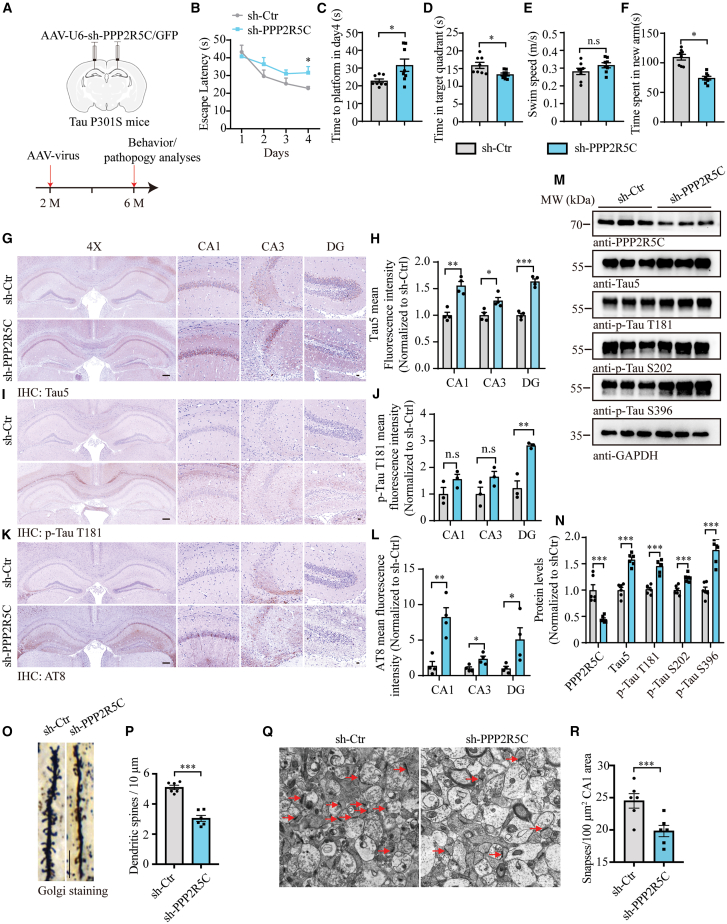
Knockdown of PPP2R5C in Tau P301S mice worsens cognitive dysfunctions (A) Schematic representation of the experimental design. Two-month-old Tau P301S mice were injected with either AAV-sh-Ctrl-EGFP or AAV-sh-PPP2R5C-EGFP. Mice were sacrificed 4 months after AAV injection. (B and C) Morris water maze analysis as escape latency (s) and escape latency on day 4 (s). (D) Probe trial performance of Morris water maze test. (E) Swim speed of mice injected with AAVs encoding sh-Ctrl-EGFP or sh-PPP2R5C-EGFP (*n* = 8–10 mice per group). (F) Time spent in the novel arm in the Y-maze test (*n* = 8–10 mice per group). (G, I, and K) Representative immunostaining images of t-Tau, p-Tau T181, and AT8 in the hippocampus of Tau P301S mice injected with AAVs encoding sh-Ctrl-EGFP or sh-PPP2R5C-EGFP. (H, J, and L) Quantifying immunoreactivity for t-Tau, p-Tau T181, and AT8 (*n* = 4 mice per group). Scale bars: 200 μm for 4× images and 20 μm for magnified images. (M and N) Representative western blots showing Tau pathology in mouse brain tissue following PPP2R5C knockdown (*n* = 6). (O) Golgi staining revealed the dendritic spines in the apical dendritic layer of the CA1 region. Scale bar, 10 μm. (P) Quantification of spine density (*n* = 6 mice per group). (Q) Electron microscopy of synapses (left) and high magnification of synapses (right). Scale bar: 2 μm in the left panel, 200 μm in the right panel. (R) Quantification of synaptic density (*n* = 6 mice per group). All the western blot data are representative of three independent experiments. Quantification data are expressed as mean ± SEM (∗*p* < 0.05, ∗∗*p* < 0.01, ∗∗∗*p* < 0.001, and n.s., no statistics, Student’s *t* test).

To further elucidate the effects of PPP2R5C in AD progression, we administered AAVs encoding mouse sh-PPP2R5C or sh-scramble with GFP tag directly into the hippocampus of 2-month-old Tau P301S mice to silent endogenous PPP2R5C expression, followed by behavioral tests after 4 months infection (Figure 5A). The Morris water maze test demonstrated that downregulating PPP2R5C accelerated spatial learning and memory of Tau P301S mice due to either significant more latency to reach the platform during the four acquisition days or less time and frequency of shuttling in target quadrant during the probe trial (Figures 5B–5E). In alignment with the Morris water maze results, AAV-shPPP2R5C-treated mice exhibited reduced exploration in the novel arm compared to sh-scramble-treated mice (Figure 5F), which reinforced the accelerated impairment of cognitive function in Tau P301S mice with endogenous PPP2R5C deficiency. Next, IHC and IF staining for the hippocampus showed heightened t-Tau (Tau5) and p-Tau (p-Tau T181 and AT8) signals in the shPPP2R5C-injected mice relative to controls with sh-scramble injection (Figures 5G–5L and S7A–S7F). Neuronal loss and the activation of microglia and astroglia were augmented after the PPP2R5C silence (Figures S7G–S7J). Similarly, we evaluated the alterations in the number of individual dendrites (Figures 5O and 5P) and the density of dendritic spines (Figures 5Q and 5R), and the results demonstrated that both of them in the hippocampus of Tau P301S mice deteriorated after the deprivation of endogenous PPP2R5C. Thus, these findings confirm that PPP2R5C plays a crucial role in the onset of AD-like pathologies in Tau P301S mice.

### PPP2R5C regulates autophagy and PP2A activity to promote Tau degradation

**Figure 6:**
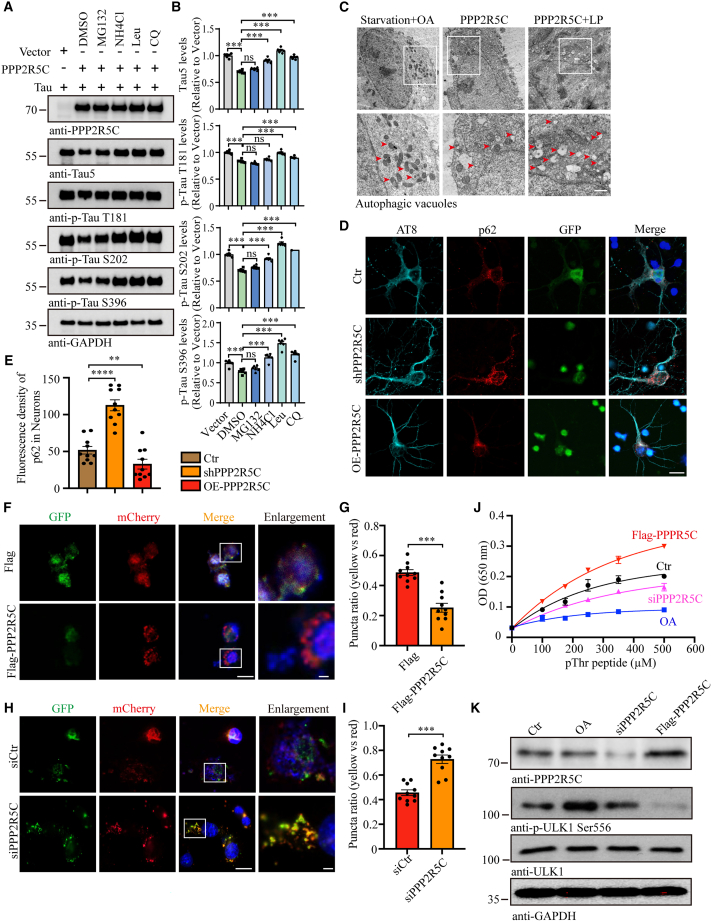
PPP2R5C promotes Tau degradation and dephosphorylation by regulating autophagy and PP2A activity (A) Representative western blots show autophagy-lysosome inhibitors NH4Cl, Leu, and CQ prevent PPP2R5C-derived Tau degradation. (B) Quantifying relative Tau, p-Tau T181, p-Tau S202, and p-Tau S396 protein levels (*n* = 6). (C) EM morphometric analysis of autophagic vacuoles in conditions of starvation combined with OA treatment and autophagic flux blockade combined with (PPP2R5C + LP) or without (PPP2R5C) treatment. Scale bar, 1 μm. (D) Representative IF images of AT8 and p62 after Tau P301S primary neurons infected with GFP-PPP2R5C/shPPP2R5C lentivirus. Scale bar, 10 μm. (E) Quantification of fluorescence density of p62 in neurons. (*n* = 10). (F and H) Representative IF images of SH-SY5Y cells co-transfected with tandem mCherry-GFP-tagged autophagy marker LC3 plus empty vector or FLAG-PPP2R5C or siPPP2R5C. Autophagosomes were visualized with yellow dots (mCherry and GFP co-localization) and autolysosomes were identified as red dots (mCherry). Scale bars: 10 μm in normal and 2 μm in Enlargement. (G and I) Quantification of puncta ratio of yellow vs. red (*n* = 10). (J) Enzymatic reaction kinetics of PP2A with different concentrations of pThr peptide. PP2A was isolated from HEK293 cells with the treatment of a PP2A inhibitor OA, FLAG-PPP2R5C, or siPPP2R5C (*n* = 6). (K) Western blot confirms the changes in PPP2R5C levels. All the western blot data are representative of three independent experiments. Quantification data are expressed as mean ± SEM (∗∗*p* < 0.01, ∗∗∗*p* < 0.001, and n.s., no statistics, Student’s *t* test).

To explore the underlying mechanisms by which PPP2R5C attenuates t-Tau levels, we utilized pharmacological inhibitors targeting different protein degradation systems. We overexpressed equal PPP2R5C and Tau through the co-transfection of FLAG-PPP2R5C and HA-Tau plasmids and then treated HEK93 cells with MG132 (proteasome inhibitor), NH4Cl (ammonium chloride, autophagy inhibitor), leupeptin ([Leu], lysosome inhibitor), or chloroquine ([CQ], autophagy/lysosome inhibitor). Immunoblotting showed that PPP2R5C-driven Tau degradation was inhibited in the presence of the autophagy-lysosome inhibitors NH4Cl, Leu, and CQ, but not by MG132 (Figures 6A and 6B), which suggests that the degradation process of Tau by PPP2R5C mainly involves the autophagolysosomal pathway.

To further confirm that PPP2R5C could initiate autophagy flux, we blocked autophagy flux by the inhibition of the lysosomal proteases cathepsins using Leu and pepstatin (LP) combined with PPP2R5C overexpression or not, parallel treatment of serum starvation plus a PP2A inhibitor okadaic acid (OA) and LP alone as two control groups. Immunoblotting showed that serum starvation strongly increased the conversion of LC3-I into LC3-II despite OA intervention. PPP2R5C overexpression also increased the ratio of LC3-II/LC-I, and blockade of the autophagic flux by LP accentuated this increase (Figures S8A and S8B). As expected, IF detection of LC3 showed that, both in the absence or the presence of LP, PPP2R5C overexpression increased the number of autophagic vacuoles ([AVs], LC3-positive vesicles) (Figure S8C). Morphometric analysis by EM clearly confirmed these observations. In the starvation plus OA group, the cells apparently underwent macroautophagy because much of the contents were encapsulated in AVs. Inhibition of cathepsins by LP led to the accumulation of vesicles with dense and compacted amorphous contents that were initiated by PPP2R5C overexpression (Figure 6C). Further, AT8 and p62 were co-stained in Tau P301S primary neurons with GFP-shPPP2R5C or GFP-PPP2R5C lentivirus infection due as p62 can interact with ubiquitinated proteins and aggresome formation can be further degraded along with ubiquitinated proteins in autolysosomes.^25^ The results demonstrated that the silence of PPP2R5C led to a marked accumulation of p62-positive protein inclusions compared to the control group, but a significant decrease in the PPP2R5C overexpression group (Figures 6D and 6E). Additionally, GFP-mCherry-LC3 staining showed a significantly increased proportion of autolysosomes and a concomitantly decreased proportion of autophagosomes in cells transfected with FLAG-PPP2R5C, contrary to the pattern of cells that silenced PPP2R5C (Figures 6F–6I). The above results suggest that PPP2R5C regulates Tau degradation by mediating the autophagolysosomal pathway.

As one of the important regulatory subunits of PP2A, the effect of PPP2R5C expression on the activity of PP2A remains unclear. Thus, we assessed the enzyme reaction kinetics of PP2A using different concentrations of pThr peptide as a substrate in HEK293 cells with PPP2R5R5C regulation and OA as a positive control. The results demonstrated that the PP2A activity was significantly augmented after PPP2R5C overexpression but was attenuated in the PPP2R5C silence group (Figures 6J and 6K). Exposure to OA led to increased phosphorylation of mTOR and AMPKα, followed by increased phosphorylation of their respective substrates, P70S6K and acetyl-CoA carboxylase (ACC).^26^ Similar effects on mTOR and AMPKα signaling pathways were obtained following PPP2R5C silencing (Figure S8D). Thus, these results indicate that PPP2R5C enhances PP2A activity to dephosphorylate Tau.

### PPP2R5C triggers autophagy through ULK1-PPP2R5C direct binding activation

**Figure 7:**
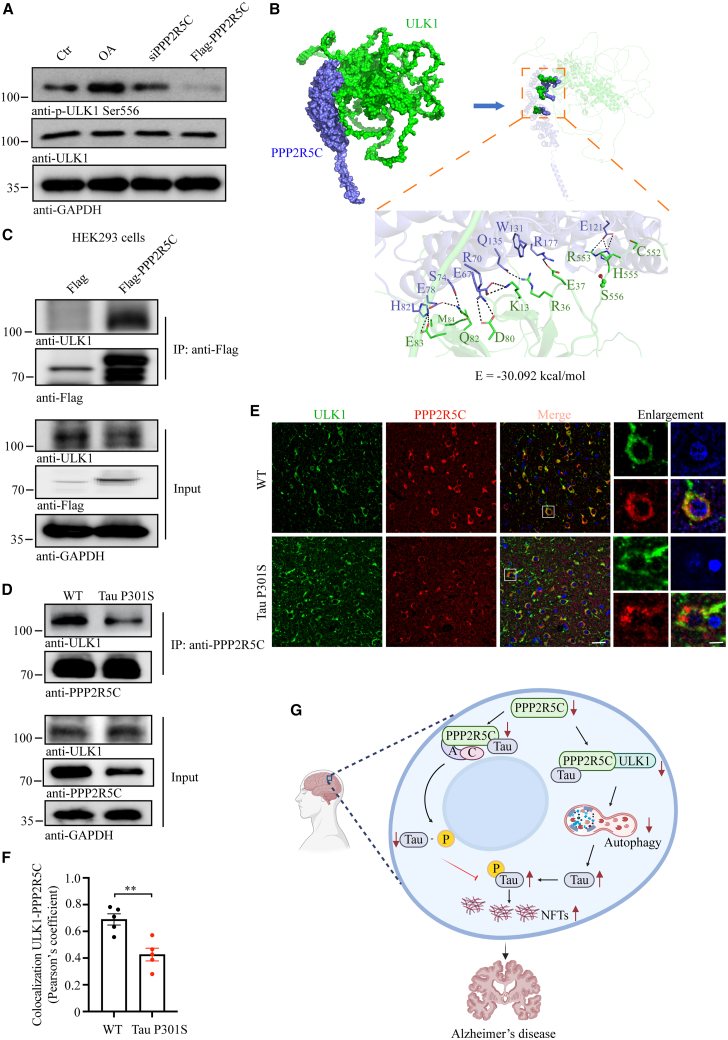
PPP2R5C triggers autophagy through ULK1-PPP2R5C direct binding activation (A) Western blot showing *p*-ULK1 Ser556 negatively correlated with PPP2R5C expression. (B) Binding positions and interaction mode analysis of proteins ULK1 and PPP2R5C. The binding interface was shown as a surface, and each protein was shown as a cartoon (right). The detailed molecular interactions of salt bridges, hydrogen bonds, and hydrophobic interactions were shown in the enlarged image, and residues on the protein interface are shown as sticks. (C) Co-immunoprecipitation assay to detect the interaction between PPP2R5C and ULK1 in HEK293 cells with FLAG-PPP2R5C or FLAG vector transfection. The co-precipitated ULK1 was subsequently detected by western blot analysis. (D) PPP2R5C and ULK1 interaction determined by co-immunoprecipitation and western blot in the cortex of WT and Tau P301S. (E) Immunofluorescence co-localization was used to observe the spatial location of PPP2R5C and ULK1 in the TauP301S and their WT littermates. Scale bars: 50 μm in normal and 20 μm in Enlargement. (F) Quantification of colocalization in (E) (*n* = 5). (G) Mechanism diagram. In the early stages of AD, PPP2R5C protein levels begin to decrease. As a PP2A enzyme catalytic subunit B family member, PPP2R5C plays a role in AD pathology by influencing Tau protein phosphorylation. Additionally, the reduction of PPP2R5C affects the ULK1-PPP2R5C-autophagy pathway, resulting in elevated total Tau (t-Tau) protein levels, further contributing to AD progression. All the western blot data are representative of three independent experiments. Quantification data are expressed as mean ± SEM (∗∗*p* < 0.01, Student’s *t* test).

Overwhelming studies have reported that ULK1 (unc-51-like kinase 1) complex regulates the early stages of the autophagy induction process.^27^^,^^28^ To elucidate whether the ULK1 activation is involved in PPP2R5C-mediated autophagy, immunoblotting results revealed that phosphorylated ULK1 was negatively correlated with PPP2R5C expression (Figure 7A). Further, a total of 100 ns molecular docking simulation time was conducted and molecular dynamics trajectory was employed for extracting the refined binding model between PPP2R5C and ULK1. The results demonstrated that the interior accessible area, mainly located at the outside of ULK1, formed the binding interface to accommodate PPP2R5C protein (Figure 7B). Polar interactions were mainly involved in stabilizing the complex via nonbonded electrostatic interactions.^29^ One highly intensive polar interaction system was formed between protein ULK1 and PPP2R5C at the interface. The binding schematic diagram for each region is shown in Figure 7B, which shows that 9 residues on ULK1 participated in forming direct interactions with 11 residues on protein PPP2R5C. Among them, 8 polar residues (H82, E78, S74, E67, R70, Q135, R177, and E121) on ULK1 formed strong salt bridges with the opposite 9 residues (E83, Q82, D80, K13, R36, E37, S556, R553, and H555) on PPP2R5C, which made the major contribution to the binding stability. Overall, the strong salt-bridge-mediated interactions (H82-E83, R70-D80, E67-K13, R177-E37, E121-R553, and E121-H555) across the binding interface seem to be pivotal to the stability of the ULK1-PPP2R5C complex. The binding affinity between ULK1 and PPP2R5C was −30.092 kcal/mol.

To further confirm the interaction between PPP2R5C and ULK1, a co-immunoprecipitaion experiment using a FLAG antibody was performed in HEK293 cells with FLAG or FLAG-PPP2R5C transfection, which demonstrated a high binding affinity between PPP2R5C and ULK1 (Figure 7C). This binding affinity, however, was attenuated in Tau P301S compared to in WT (Figure 7D), which is in alignment with significantly decreased co-localized fluorescence intensity of PPP2R5C and ULK1 (Figures 7E and 7F). This evidence suggests that PPP2R5C directly binds ULK1, providing a favorable condition for ULK1 dephosphorylation to initiate autophagy. The mechanism diagram further illustrates the mechanistic effects of PPP2R5C downregulation in AD pathology, consisting of two major pathological mechanisms. First, decreased PPP2R5C expression reduces PP2A enzyme activity, increasing p-Tau levels and exacerbating AD pathology. Second, the reduced interaction between PPP2R5C and ULK1 leads to diminished autophagy activation. This suppression of autophagy inhibits Tau protein degradation, causing Tau accumulation in neuronal cells, further aggravating AD pathology (Figure 7G).

## Discussion

Currently, AD has emerged as one of the most significant challenges affecting tens of millions of people worldwide and threatening the health of older adults.^30^ Early intervention has been a critical strategy for delaying the progression of AD, as pharmacological treatments have limited efficacy in the dementia stage.^31^ Thus, there is an imperative need for superior early warning patterns and diagnostic biomarkers to support early diagnosis of AD and clarify the regulatory mechanisms involved. To our knowledge, our study is the first to report that PPP2R5C could be an ideal biomarker for early screening of AD. By employing a diverse set of analytical methods and incorporating both human and animal data, we provide convergent lines of evidence that strengthen the validity of our conclusions, thereby increasing the robustness of our findings and enhancing the translational relevance of PPP2R5C.

The development of early diagnostic markers for AD based on blood has become a general consensus. Blood biomarkers are intrinsically beneficial since blood draws are less intrusive and can be conducted repeatedly for early-stage diagnosis, monitoring of disease progression, and assessment of therapeutic response.^8^ There are two key challenges in developing blood biomarkers for AD. One is to achieve specificity for changes occurring in neurons or other brain cells rather than non-neuronal sources.^32^ The other challenge is that the biomarkers cross the blood-brain barrier (BBB) smoothly and remain stable in the blood.^33^ The PPP2R5C we identified is originally from NDEs, characterized by nanosized particles surrounded by a lipid bilayer membrane, which facilitates the smooth passage of its contents through the BBB into peripheral blood circulation. Meanwhile, our results clearly demonstrated that PPP2R5C was specifically expressed in neurons, which may provide a molecular snapshot of the neuronal situation in the brain. Besides, it is worth emphasizing that the change of PPP2R5C levels in plasma can be synchronized with its alteration in NDEs, which completely overcomes the limitation that NDEs are difficult to enrich.

In this study, we utilized five cohorts to identify and validate PPP2R5C changes during the AD progression and further test its diagnostic efficacy. Plasma PPP2R5C levels were significantly lower in the aMCI group than in the healthy control group, demonstrating significant efficacy in the early diagnosis of AD, which was consistent with our findings in NDEs. Meanwhile, the diagnostic efficiency of plasma PPP2R5C for AD and MCI has reached 0.8494 and 0.7360, respectively, under the ELISA-based method. We believe that it will perform more excellently under a detection platform with higher detection accuracy. Additionally, it is noteworthy that PPP2R5C still exhibited good differential diagnostic performance in distinguishing AD from PSP and FTD, despite all three conditions having tauopathy. The difference is that the vast majority of PSP and FTD patients are a 4R-tauopathy, predominated by subcortical pathology in neurons, astrocytes, and oligodendroglia,^34^^,^^35^ while NFTs in AD are composed of 3R and 4R Tau isoforms in paired helical filaments.^36^ Combined with the mechanism investigation in our study, whether PPP2R5C has a different affinity to 3R and 4R Tau is worth further exploration. Meanwhile, we did not see a reduction in PPP2R5C in the normal elderly population, suggesting that the aging factors may not be sufficient to affect PPP2R5C expression. The more exciting aspect is that the decrease in PPP2R5C seems to occur prior to the hyperphosphorylation of Tau protein, which is fully consistent with our subsequent elucidation of the mechanism by which PPP2R5C affects total Tau and phosphorylated Tau. The above analysis suggests a more promising potential value of PPP2R5C as a specific biomarker for early diagnosis of AD. However, why PPP2R5C gradually decreases during AD progression is also an issue worth exploring.

PP2A core enzyme consists of a 65-kDa scaffold subunit (A subunit) and a 36-kDa catalytic subunit (C subunit).^37^ To achieve full activity against certain substrates, the PP2A core enzyme interacts with a variable regulatory component (B subunit) to form a heterotrimeric holoenzyme.^38^ PPP2R5C, known as B56β, is the second member in the regulatory subunit B' (also known as B56 or PR61), which is most highly expressed in the brain compared to other members of its family, such as B56α, B56γ, and B56δ.^39^ PPP2R5C interacts with chicken acidic leucine-rich epidermal growth factor-like domain-containing brain protein/neuroglycan C (CALEB/NGC) and inhibits CALEB/NGC-mediated dendritic branching, but not spine formation.^40^ Mutations in PPP2R5C were associated with intellectual disability and neurodevelopmental delay.^41^ These previous reports have demonstrated the crucial role of PPP2R5C in the nervous system. Our studies, both *in vivo* and *in vitro*, have found that PPP2R5C binds to Tau, reducing the expression of total Tau and dephosphorylating Tau, further confirming the vital role of PPP2R5C in neurons. In addition, PP2A is responsible for approximately 70% of Tau phosphatase activity, likely due to the high affinity of PPP2R5C for Tau, which enables this heterotrimeric enzyme to dephosphorylate Tau efficiently. Thus, it is worthwhile to investigate the Tau-binding elements in PPP2R5C and the dephosphorylation pattern of Tau by PP2A containing PPP2R5C at the molecular level.

Autophagy is a critical degradation system in mammalian cells that clears aberrant protein aggregates and maintains protein homeostasis and neuronal health.^42^ Several studies have found that autophagy deficiencies occur in the early stages of AD.^43^ Previous research has indicated that the regulation of PP2A activity during autophagosome formation is complex and diverse. PP2A can modulate the phosphorylation of a wide range of substrates, some of which, such as mTOR/ULK pathway components, are molecular targets upstream of the autophagy core machinery.^44^ It is worth noting that an increase in mTOR phosphorylation and activity was documented in AD brains.^45^ In mammalian cells, mTORC1 phosphorylates ULK1 at Ser758, thereby blocking the interaction and subsequent phosphorylation of ULK1 by AMPK, which is required for ULK1 activation.^46^ In our study, we first confirmed that mediating PPP2R5C can positively affect PP2A activity. Knocking down PPP2R5C activates the mTOR/ULK1/P70S6 and AMPKα/ACC pathways, suggesting that PPP2R5C regulates autophagy dependent on its effect on PP2A activity. Our findings are consistent with a previous report that PP2A upregulation by PP2A catalytic subunit (PP2Ac) overexpression stimulates neuronal autophagy.^47^

The initiation and formation of an autophagosome is a sophisticated process involving the very first autophagy-specific ULK1 complex.^28^ The essentiality of ULK1-dependent autophagy relies on the interaction between ULK1 and its binding partners, such as ATG13, FIP200, and ATG101.^48^ However, it seems that the ULK1 interaction network is significantly more intricate and encompasses a multitude of distinct and varied proteins. In our study, we found that PPP2R5C interacts with ULK1, and this interaction was significantly reduced in the brains of Tau P301S mice compared to their age- and sex-matched WT littermates. Together with previous studies on autophagy impairment either in Tau P301S mice or AD patients to trigger Tau aggregation, there is every reason to believe that PPP2R5C influences Tau metabolism by binding to ULK1 alone to affect neuronal autophagy.

In summary, for the first time, we propose that plasma PPP2R5C has the potential to serve as an excellent biomarker with high efficacy for the early screening of AD. Furthermore, substantial evidence suggests that PPP2R5C relies on the PP2A enzyme to regulate the levels of phosphorylated Tau and autophagy. Alternatively, PPP2R5C can independently modulate autophagy to influence the total Tau in neurons. The successful elucidation of the intrinsic mechanism provides a reliable guarantee for PPP2R5C as a biomarker for the early diagnosis of AD.

### Limitations of the study

Despite the strengths of our study, several limitations should be acknowledged. First, although we included multiple independent cohorts and validated PPP2R5C in both human samples and animal models, the sample sizes within some subgroups remain relatively modest, which may limit the generalizability of the findings. As the discovery cohort of biomarkers, cohort 1 employed the in-group mixing method for proteomic determination, which may have obscured some key information to some extent, although we conducted additional biological verification for the discovery of PPP2R5C. Second, the sensitivity of PPP2R5C is relatively low in terms of diagnostic efficacy, which may be due to the ELISA not meeting the requirements for detecting low abundance. As a declining biomarker, there is an urgent need to develop more sensitive assays, such as single-molecule immunoassays, to improve the high sensitivity of PPP2R5C for early diagnosis of AD. Third, while our data strongly support the diagnostic potential of PPP2R5C, further large-scale, multicenter studies are necessary to confirm its clinical utility across diverse populations. Finally, as with perfect biomarker studies, longitudinal analyses will be essential to establish whether changes in PPP2R5C reliably predict disease onset and progression over time. Addressing these limitations in future work will be critical to fully realize the translational potential of PPP2R5C as a biomarker for early AD diagnosis.

## Resource availability

### Lead contact

The resources, information, reagents, and data supporting the findings of this study are available upon reasonable request to the lead contact, Lu Shen (shenlu@csu.edu.cn).

### Materials availability

This study did not generate new unique reagents. All reagents used in the study are commercially available or obtained from publicly accessible sources.

### Data and code availability

• The mass spectrometry proteomic data have been deposited to the ProteomeXchange Consortium (https://proteomecentral.proteomexchange.org) via iProX partner repository with the dataset identifier PXD072740.
• This study did not generate code.
• Study data are available with the article and its supplemental information. Any additional information required to reanalyze the data reported in this paper is available from the lead contact upon request.

## Acknowledgments

This research was supported by the STI2030-Major Projects (2021ZD0201803), the 10.13039/501100001809National Natural Science Foundation of China (U22A20300, 81971029, and 82371434), the Scientific Research Program of FuRong Laboratory (2024PT5108), and the Outstanding Youth Fund of Hunan Provincial Natural Science Foundation (2025JJ20100 and 2024JJ2097). We gratefully acknowledge the patients who participated in this study. All participants provided written informed consent.

## Author contributions

S.L. and H.L. contributed equally to this work. S.L. wrote the manuscript. H.L. and S.L. designed and performed most of the experiments. T.X. and X. Liu performed the LC-MS/MS experiments. Y. Li performed the ELISA assays. X.X., X. Liao, and Y. Liu assisted with data analysis. Y.Z., J.-L.W., J.G., and B.T. contributed to clinical sample collection. X.Y. collected samples of human brain tissue. B.J., Z.Z., and L.S. conceived the project and critically read the manuscript.

## Declaration of interests

The authors declare no competing interests.

## STAR★Methods

### Key resources table

**Table:** 

REAGENT or RESOURCE	SOURCE	IDENTIFIER
**Antibodies**		

CD171	Abcam, #ab270455	RRID:AB_2893221
Alix	Abcam, #ab275377	RRID:AB_3644262
PPPR5C	Thermo, #PA5-110209	RRID:AB_2855620
Tau5	Thermo, #13-6400	RRID:AB_2533025
p-Tau T181	Thermo, #701530	RRID:AB_2532491
p-Tau S202	Abcam, #ab108387	RRID:AB_10860874
p-Tau S396	Thermo, #44-752G	RRID:AB_2533745
AT8	Thermo, #MN1020	RRID:AB_223647
*p*-ULK1 Ser556	Proteintech, #80218-1-RR	RRID:AB_2918877
ULK1	Proteintech, #20986-1-AP	RRID:AB_2878783
Flag	Proteintech, #20543-1-AP	RRID:AB_11232216
LC3	Cell Signaling Technology, #12741	RRID:AB_2617131
mTOR	Cell Signaling Technology, #2983	RRID:AB_2105622
*p*-mTOR	Cell Signaling Technology, #2971	RRID:AB_330970
*p*-AMPKα	Cell Signaling Technology, #2535	RRID:AB_331250
AMPKα	Cell Signaling Technology, #2603	RRID:AB_490795
p-P70S6K	Proteintech, #28735-1-AP	RRID:AB_2918197
P70S6K	Proteintech, #14485-1-AP	RRID:AB_2269787
*p*-ACC	Cell Signaling Technology, #11818	RRID:AB_2687505
ACC	Cell Signaling Technology, #3676	RRID:AB_2219397
p62	Cell Signaling Technology, #23214	RRID:AB_2798858
Synaptophysin	Cell Signaling Technology, #36406	RRID:AB_2799098
Trem2	Abcam, #ab209814	RRID:AB_3095849
GLAST	Proteintech, #20785-1-AP	RRID:AB_2878738
GAPDH	Proteintech, #60004-1	RRID:AB_2107436

**Bacterial and virus strains**		

Stellar™ Competent E. coli (High Efficiency)	Takara, #636763	N/A
AAV2/9	BrainVTA (Wuhan) Co., Ltd	N/A

**Biological samples**		

Plasma/NDEs samples	Xiangya Hopsital	N/A
Brain tissues	Xiangya Human Brain Bank of Central South University	This paper

**Chemicals, peptides, and recombinant proteins**		

Formic acid	Sigma	Cat# 507
Acetonitrile	Thermo	Cat# 047138.K2
Trypsin	Thermo	Cat# 25200072
Neurobasal medium	Gibco	Cat# 21103049
Gibco B-27	Gibco	Cat# 17504044
Lipofectamine 3000	Invitrogen	Cat# L3000075
opti-MEM	Invitrogen	Cat# 31985070
DiI powder	Invitrogen	Cat# D282
pNPP Ser/Thr assay buffer	Sigma	Cat# 20-179
threonine phosphopeptide	Sigma	Cat# 12-219
Phosphate standard	Sigma	Cat# 20-103

**Critical commercial assays**		

IHC Detection System Kit	Absin	Cat# abs957
Golgi staining Kit	FD Neuro Technologies, Inc.	Cat# PK40
Pierce™ BCA protein assay kit	Thermo	Cat# 23227
PPP2R5C ELISA kit	Abbexa	Cat# abx500105
Protein A + G-agarose beads	Beyotime	Cat# P2012
Lipofectamine 3000	Invitrogen	Cat#L3000075

**Deposited data**		

Proteomic data	This paper	iProx: IPX0015046000
UniProt human protein database	UniProt human protein	https://www.uniprot.org

**Experimental models: Cell lines**		

SH-SY5Y	Cellosaurus	RRID:CVCL_0019
HEK293	Cellosaurus	RRID:CVCL_0045

**Experimental models: Organisms/strains**		

C57BL/6J mice	Jackson Labs	Cat# 000664
Tau P301S mice	Jackson Labs	Cat# 008169

**Software and algorithms**		

SnapGene	SnapGene	7.2.1
GraphPad Prism	GraphPad	9.0
R software	The R Project for Statistical Computing	www.r_project.org
ANY-Maze tracking software	San Diego Instruments	6.2
AMBER software	AMBER	version 16
Rosetta	Rosetta	3.1

### Experimental model and study participant details

#### Study participants

In this study, participants were recruited from the Department of Neurology at Xiangya Hospital of Central South University between 2016 and 2023, as well as from the Jili cohort in Liuyang, Hunan Province. Patients with AD, amnestic mild cognitive impairment (aMCI), met the 2011 National Institute on Aging and the Alzheimer’s Association (NIA-AA) criteria for AD and aMCI, and patients with Frontotemporal dementia (FTD), and progressive supranuclear palsy (PSP) were diagnosed according to the FTD and MDS-PSP diagnostic criteria, respectively.^49^^,^^50^ The clinical and demographic features of the diagnostic cohorts are shown in Table S1. The study protocol was approved by the Institutional Review Board of Xiangya Hospital of Central South University in China (2019030501). Written informed consent was obtained from each participant or guardian.

Cohort 1 consists of 41 participants, comprising 13 patients with familial AD (FAD), 10 individuals with presymptomatic FAD (stage 0, according to 2024 NIA-AA criteria), and 18 cognitively normal controls. All FAD and preFAD participants carried pathogenic mutations in the *APP*, *PSEN1*, or *PSEN2* genes, determined using gene-targeted sequencing.^51^ We used cohort 1 to discover the distinctive NDEs index in plasma. Cohort 2 was used to validate the PPP2R5C tendency in NDEs, which consisted of 64 subjects, including 20 patients with mild sporadic AD (SAD), 12 with aMCI, and 32 age- and gender-matched cognitively normal controls. Cohort 3 was an independent validation FAD cohort from Collaborative Network in China (CI-MDCNC), comprising 15 FAD patients and 15 cognitively normal controls. Cohort 4 aimed to detect the PPP2R5C level in plasma and assess its diagnostic ability for AD, including 74 Aβ-positive AD cases, 76 with Aβ-positive aMCI, and 74 cognitively normal controls. All AD and aMCI were Aβ positive and validated by Aβ-PET or CSF biomarkers. Cohort 5 aimed to clarify differential efficiency, comprising 34 patients with AD, 37 with FTD, and 28 with PSP.

#### Human brain tissue sample

Postmortem human brains were banked through a willed body donation program with the donor’s clinical information retrieved before and/or during the previous hospitalization, as available.^52^ All brains were histopathologically treated using the Standard Brain Banking Protocol established by the China Brain Bank Consortium.^53^ AD cases were clinically diagnosed and neuropathologically confirmed. The average ages of the young, aged, and aged AD patients were 17.6 (*n* = 4, 2 females and 2 males), 66.9 (*n* = 7, 4 females and 3 males), and 65.4 (*n* = 7, 5 females and 2 males), respectively. The brains were dissected and preserved in a fresh-frozen state at −70°C until use. The healthy and Braak-graded AD brain slices (Table S3) were obtained from Xiangya Human Brain Bank, Central South University. Human samples were obtained with the approval of the Ethics Committee and written informed consent.

### Method details

#### Neuron-derived exosome isolation

Ten milliliters of venous blood were collected from fasting participants (overnight, 8–12 h) into EDTA-prepared polypropylene tubes. Each sample was promptly centrifuged at 2,000 × g for 10 min at 4°C within 2 h of collection. For each sample, 0.5 mL of the obtained plasma was first treated with protease and phosphatase inhibitors to prevent degradation. Subsequently, 5 μL of Thrombin Plasma Prep was introduced into the plasma for exosome precipitation. This mixture was then centrifuged at 10,000 × g for 5 min. The supernatant obtained was mixed with 10 μL Pierce Streptavidin Agarose Resin and 3% BSA. The mixture was incubated at 4°C for 5 h, followed by centrifugation at 2,500 × g for 10 min. The supernatant was then collected for further processing. The supernatant was incubated with Exosomes Precipitation Solution (EXOQ; System Biosciences, CA) on ice for 1 h, followed by a centrifugation step at 1,500 g for 30 min. The pellet was retained and resuspended in 250 μL of PBS, along with additional protease and phosphatase inhibitors. For neuron-specific exosome targeting, the resuspended pellet was incubated with 4 μL of anti-human CD171-biotin and 25 μL of Pierce Streptavidin Agarose Resin overnight at 4°C. After overnight incubation, samples underwent final centrifugation at 200 × g for 10 min at 4°C. The supernatant was discarded, and the pellet was vortexed for 10 s in 0.05 M glycine-HCl (pH 3.0). We added 0.45 mL of PBS containing 2% BSA, 0.10% Tween 20, and inhibitor cocktails to the pellet. The sample was incubated at 37°C for 10 min with intermittent vortexing. Post-incubation, the sample was stored at −80°C for subsequent analysis.

#### Transmission electron microscopy

NDEs were visualized using TEM as previously described.^54^ Briefly, the NDEs were resuspended in glycine-HCl (pH 3.0), and the pH was adjusted to neutrality (pH 7.0) using Tris-HCl (pH 8.6). TEM analysis was performed following a standardized protocol.^55^ NDEs were identified based on their size, ranging from 30 to 150 nm, and their distinctive morphology—rounded shapes with cup-shaped profiles and a bilayer lipid membrane. Synaptic density was evaluated using electron microscopy following previously established methodologies.^56^ Mice were deeply anesthetized and underwent transcardial perfusion with 2% glutaraldehyde. Subsequently, the hippocampal regions were shielded from light and maintained at room temperature for two hours, followed by an overnight incubation at 4°C. Tissue sections were then postfixed with 1% OsO4 at cold temperatures for one hour. For imaging, mouse samples were processed following routine procedures. Ultrathin sections (90 nm thick) were contrast-enhanced with uranyl acetate and lead acetate. The prepared samples were examined with a JEOL 200CX electron microscope operating at an accelerating voltage of 100 kV. Synaptic integrity was assessed by the clear visualization of synaptic vesicles and postsynaptic densities, indicative of active synapses.

#### Nanoparticle tracking analysis

The concentration and size distribution of NDEs were quantified using NTA with a ZetaView PMX 110 (Particle Metrix, Germany) equipped with a 488 nm laser, following previously established methods.^57^ Briefly, samples were diluted in PBS to reach a final volume of 1 mL. Prior testing determined the optimal concentrations yielding 20–100 particles per frame to ensure accurate tracking. Measurements were conducted across 11 distinct cell positions within the sample chamber. Captured videos were subsequently processed using the integrated ZetaView software. Analysis parameters were set as follows: maximum particle size at 1,000 nm, minimum particle size at 10 nm, and a minimum brightness threshold at 30 units.

#### NDEs protein extraction and trypsin digestion

The NDEs were lysed in TCEP buffer, which comprises 2% deoxycholic acid sodium salt, 40 mM 2-chloroacetamide, 100 mM Tris-HCl, 10 mM Tris (2-chloroethyl) phosphate, and 1 mM PMSF, mixed with MS water at pH 8.5, and subsequently heated in a metal bath at 99°C for 30 min. Following cooling to room temperature, trypsin was added, and digestion proceeded for 17 h at 37°C. 10% FA was added to each tube, vortexed for 3 min, and subsequently centrifuged at 12,000 g for 5 min. The supernatant was transferred to a new 1.5 mL tube and subsequently dried using a vacuum drier at 60°C. Then, the peptides were dissolved in 100 μL of 0.1% FA, vortexed for 3 min, and then sedimented for 5 min (12,000 × g). The supernatant was transferred to a new tube and subsequently desalinated. Prior to desalination, the pillars must be activated with two 3M C18 disk inserts. The lipid is administered in the following order: 90 mL of 100% ACN twice, 90 mL of 50% and 80% ACN once, and then 90 mL of 50% ACN once. The supernatant from the tubes was loaded into the pillar twice after the pillar was balanced with 90 mL of 0.1% FA, and then decontaminated with 90 mL of 0.1% FA twice. Finally, the effluent was collected for MS after 90 mL of elution buffer (0.1%FA in 50% ACN) was added to the pilar for elution twice. The collected peptides were subsequently desiccated at 60°C using a vacuum dryer.

#### Label-free LC-MS/MS detection

The acquisition of samples was randomized to avoid bias. Proteomic profiling was conducted on a Q Exactive HF-X Hybrid Quadrupole-Orbitrap mass spectrometer (Thermo Scientific), integrated with an Easy nLC 1200 system (Proxeon Biosystems). The dried peptides were reconstituted in 40 μL of 0.1% (v/v) formic acid. An equal amount of 1 μg per sample was loaded onto a reverse-phase trap column (Thermo Scientific Acclaim PepMap 100 μm∗2 cm, nanoViper C18) connected to the C18-reversed phase analytical column (Thermo Scientific Easy Column, 10 cm long, 75 μm inner diameter, 3 μm resin) with a gradient of 5–55% (0–110 min) and 55–100% (110–115 min) mobile phase B (ACN with 0.1% FA) at a flow rate of 300 nL/min. The mobile phase A was water with 0.1% FA. The eluted peptides underwent ionization and detection via high-field asymmetric waveform ion mobility spectrometry in conjunction with QE-HF-X (Thermo Scientific). The DV was established at −30V. Mass spectrometry was conducted using data-dependent acquisition (DDA) mode. The MS1 Spectra full scan involved the acquisition of ions with *m/z* values between 300 and 1,800 using a QE-HF-X mass analyzer, achieving a high resolution of 70,000. MS2 spectral acquisition was performed in the ion trap in a rapid speed mode. Fragmentation was performed via High Collision Dissociation (HCD), with an Automatic Gain Control (AGC) target set at 1e6 ions to maintain consistent signal intensity. The maximum injection time was limited to 50 ms to optimize ion sampling. Dynamic exclusion was programmed at 30 s to prevent the repetitive sampling of previously fragmented ions. Survey scans reached a high resolution of 70,000 at *m/z* 200, ensuring precise mass determinations. For HCD spectra, the resolution was set to 17,500 at *m/z* 200, striking a deliberate balance between analytical speed and detail. Ions were isolated with a 1.5 *m/z* window, enabling accurate mass selection for fragmentation. The normalized collision energy was carefully set to 30% to ensure efficient fragmentation without compromising integrity. The underfill ratio was sustained at 0.1%, guaranteeing an optimal ion count per scan. The peptide recognition mode was engaged throughout the run to enhance the accuracy of peptide identification.

#### Peptide identification and protein quantification

The MS raw data for each sample were combined and searched using the MaxQuant 1.3.0.5 software for identification and quantitation analysis. The main software parameters were set as follows: the enzyme is trypsin, the maximum missed cleavages is 2, the fixed modification is carbamidomethyl (C), and the dynamic modification is oxidation (M). The central search tolerance is 6 ppm, the first search tolerance is 20 ppm, and the MS/MS tolerance is 20 ppm. The data were searched against the UniProt human protein database (updated on 2019.12.17, 20406 entries) with the database pattern set as Reverse. Contaminants are included in the search. All reported data were based on 99% confidence for both protein and peptide identification, as determined by false discovery rate (FDR) ≤ 0.01.

#### Quality control of the mass spectrometry data

Quality control of the label-free DDA MS/MS data was evaluated based on peptide identification scores and mass accuracy. The high median peptide score (122.51) and the fact that 95.49% of peptides had scores above 60 indicated reliable peptide identification in NDEs (Figure S1A), while the mass error distribution centered around 0 ppm within ±5 ppm demonstrated excellent mass accuracy and instrument stability (Figure S1B). In addition, the distribution of protein expression ratios in the NDEs group analysis was roughly symmetrical around zero, with the range of fold changes within reasonable limits, suggesting balanced data and reliable quantitative comparisons between samples (Figure S1C). The protein coverage distribution data are shown in Figure S1D, which follows the typical pattern observed in label-free proteomics. Proteins identified in more than half of the samples in each group were selected for the calculation of the coefficient of variation (CV). The median CV value was 2.3% in the C group, 2.9% in the P group, and 1.6% in the Pre group, indicating good intra-group sample reproducibility.

#### Targeted PRM analysis

To validate and quantify the target proteins, we established a parallel reaction monitoring (PRM) strategy. For method development, randomly selected plasma samples (20 μg protein each) were digested using trypsin following the filter-aided sample preparation (FASP) protocol. The digested peptides were desalted, lyophilized, and reconstituted in 0.1% FA, and then quantified by OD280. Based on the sequences of 26 target proteins and the results from label-free analysis, specific peptides unique to the target proteins were selected for subsequent PRM validation. Shotgun LC-MS/MS analysis was performed, followed by transition selection in Skyline to identify reliable peptides and fragment ions suitable for PRM quantification.

For PRM method optimization, pooled peptide samples were spiked with AQUA internal standard peptides and analyzed by LC-RPM/MS in triplicate. Optimized conditions were then applied to all validation samples. In the formal PRM assays, 2 μg peptide digest from each sample was spiked with 10 fmol of a heavy isotope-labeled reference peptide (DSPSAPVNVTVR). Peptides were separated on an Easy-nLC system at a flow rate of 250 nL/min using a 60 min gradient (5–100% buffer B, 0.1% formic acid in 84% acetonitrile). The Q Exactive mass spectrometer (Thermo Scientific) was operated in positive ion mode with a full MS scan at a resolution of 60,000 (at 200 *m/z*), AGC target of 3e6, maximum injection time of 200 ms, and a scan range of 300–1800 *m/z*. Target precursors were isolated with a 1.6 Th window and fragmented by HCD at 27% normalized collision energy. MS2 spectra were acquired at a resolution of 30,000 (at 200 *m/z*) with a maximum injection time of 120 ms. The Raw files were analyzed using Skyline (version 3.5.0) with a cutoff score of 0.95. Candidate transitions were verified according to retention time, mass accuracy, and MS/MS spectra. Only peptides with stable signals and well-defined peak shapes were retained for final quantification.

#### ELISA

Plasma PPP2R5C protein levels were measured using a commercially available ELISA kit (Abbexa, #abx500105). Briefly, plasma samples were diluted according to the manufacturer’s instructions and added to 96-well plates pre-coated with capture antibodies specific for PPP2R5C. After incubation, plates were washed to remove unbound material, and a biotinylated detection antibody was added to each well. Following further incubation and washing, streptavidin-conjugated horseradish peroxidase (HRP) was applied. The signal was developed using a tetramethylbenzidine (TMB) substrate, and the reaction was stopped with sulfuric acid. The optical density (OD) was measured at a wavelength of 450 nm. The concentration of PPP2R5C in each sample was compared with a standard curve generated from known concentrations of recombinant PPP2R5C.

#### Plasma biomarker detection

Venous blood samples were collected in EDTA tubes and centrifuged within two hours of collection to obtain plasma samples. The plasma samples were stored at −80°C. Quantification of Aβ42, Aβ40, and phosphorylated tau (p-tau181, p-tau217, p-tau231) was performed using a fully automated single-molecule detection system (AST-Sc-Lite, AstraBio) according to the manufacturer’s protocols.^49^ Briefly, plasma samples were diluted and incubated with paramagnetic beads coated with specific capture antibodies for the target analytes. The beads were then washed and incubated with biotinylated detection antibodies. Following a second washing step, streptavidin-β-galactosidase was added to generate a fluorescent signal. The beads were resuspended and transferred to the disc array, where individual beads were isolated in microwells. The fluorescence signal from each bead was detected and quantified, allowing for ultra-sensitive detection of the target proteins. Concentrations were determined using a calibration curve derived from serial dilutions of known standards. The assay kits used were R64020, R64010, R64030, R64050, and R64100 (Suzhou AstraBio Technology), which were specifically designed for detecting plasma biomarkers of Aβ42, Aβ40, p-tau181, p-tau217, and p-tau231, respectively. The personnel conducting the tests were blinded to the participants’ group status.

#### Primary neuron culture

Primary neurons were harvested from embryonic day 18 Tau P301S mice and cultured in a Neurobasal medium enriched with B27 supplement, following established protocols.^58^ At five days *in vitro* (DIV 5), neurons were transfected with Lentivirus (Lv). The specific constructs used were GFP-Vector, GFP-PPP2R5C, GFP-sh-Scramble, or GFP-sh-PPP2R5C. After one week post-transfection, neurons underwent fixation in 4% formaldehyde. They were then permeabilized and immunofluorescence staining using primary antibodies targeting Tau5, p-Tau181, p62, AT8, and GFP, respectively. Treated sections were mounted with a glass coverslip using a standard mounting medium. Visualization and analysis were conducted to assess protein expression and neuronal morphology.

#### Western blotting

To prepare whole-cell lysates, cultured cells, and mouse brain tissues were lysed with RIRA buffer (NCM, #WB3100) containing a mixture of protease and phosphorylase inhibitors on ice for 15 min. Lysates were centrifuged at 14,000 rpm for 15 min at 4°C, and then the supernatant was collected. Protein concentration was measured by BCA assay, and an equal amount of protein was loaded for blotting.

#### Co-immunoprecipitation

HEK293 cells were co-transfected with plasmids encoding Flag-tagged PPP2R5C and HA-tagged Tau or the corresponding empty vector. Cells were harvested 48 h post-transfection for subsequent analyses. The cells were lysed using IP lysis buffer (Beyotime, catalog no. P0013F) for one hour at 4°C. Cell debris was removed by centrifugation at 15,000 × g for 5 min. The clarified lysates were incubated with mouse monoclonal anti-Flag and anti-HA antibodies overnight at 4°C to form antigen-antibody complexes. Protein A + G-agarose beads (Beyotime, catalog no. P2012) were then added to the lysates, and the incubation continued for an additional 3 h at 4°C. Following binding, the beads were washed five times with the lysis buffer to remove non-specifically bound proteins. Samples were then eluted by adding a loading buffer and boiling for 5 min to release the immunocomplexes from the beads. The eluted samples were separated using SDS-PAGE and then transferred to membranes for WB. The immunoblots were probed to detect the presence of HA-tagged and Flag-tagged proteins.

#### Immunostaining

Mouse brain sections embedded in paraffin were deparaffinized, rehydrated, and subjected to antigen retrieval using 0.1 M sodium citrate buffer (pH 6.0) at 94°C for 20 min. Endogenous peroxidase activity was quenched with a 3% H_2_O_2_ treatment for 10 min to minimize nonspecific background. Sections were blocked with 3% BSA and 0.3% Triton X-100 for 30 min to prevent non-specific binding. Overnight incubation at 4°C with primary antibodies against Tau5, AT8, and p-Tau T181 followed. Post-primary incubation and sections were thoroughly rinsed with PBS. For immunohistochemistry, signal amplification was conducted per the High-Efficiency IHC Detection System Kit (Absin, #abs957). For immunofluorescence double-staining, Alexa Fluor 488- and 594-conjugated secondary antibodies were applied. For immunocytochemistry (ICC) for cultured neurons, neurons were fixed with 4% paraformaldehyde and 0.1% Triton X-100 for 15 min, washed, and then blocked with 3% BSA for 30 min. Primary antibodies against tau5, AT8, and p-T181-tau were incubated overnight at 4°C. After the primary antibody application, neurons were incubated with Alexa Fluor 488- and 594-conjugated secondary antibodies for 2 h at room temperature in the dark. Following secondary antibody application, cells were stained with DAPI for nuclear visualization and examined under a fluorescence microscope after final PBS washes.

#### Cells and transfection

HEK293 and SH-SY5Y cells were cultured in high-glucose DMEM or DMEM/F12 supplemented with 10% FBS and penicillin (100 units/mL)/streptomycin (100 μg/mL). The Flag-PPP2R5C plasmid was modified from Flag/ZsGreen-PPP2R5C, siRNA PPP2R5C was purchased from Santa Cruz Biotechnology (#sc-45847). HEK293 and SH-SY5Y transfection was performed using Nano293T (NCM, #C500T-1) and Lipofectamine 3000 (Invitrogen, #L3000075), respectively, according to the manufacturer’s protocol with opti-MEM (Invitrogen, #31985070) as transfection solution. Transfected cells were incubated at 37°C for 48 h and then harvested for further experiments.

#### DiI staining

DiI staining was used to illustrate the dendritic density of neurons. Neurons treated with adeno-associated viruses (AAVs) were fixed in 4% paraformaldehyde for 15 min. After fixation, the neurons were gently rinsed with PBS to remove residual fixatives, preparing them for optimal dye penetration. DiI powder (Invitrogen, #D282), chosen for its affinity for neuronal membranes, was applied uniformly across the neuronal slices. The slices were left undisturbed for 20 min, allowing the lipophilic dye to diffuse adequately through the dendritic. Then, slices were thoroughly rinsed with PBS to wash away unbound DiI, minimizing non-specific background fluorescence. The stained slices were mounted on microscope slides and examined with the confocal laser microscope (LEICA). This imaging allowed for the high-fidelity visualization of dendritic density, specifically focusing on the labeled dendritic processes.

#### Stereotaxic injection

The coding sequence of human PPP2R5C or sh-PPP2R5C was inserted into hSyn promoter AAV (GFP tag) and U6 promoter AAV (GFP tag), respectively. The virus was packaged by BrainVTA (Wuhan) Co., Ltd. Three-month-old Tau P301S mice, regardless of gender, were anesthetized with isoflurane (Piramal Healthcare). Meloxicam (2 mg/kg) was injected subcutaneously as an analgesic (Loxicon, Norbrook). Bilateral intracerebral injection of AAV-GFP-PPP2R5C (titer: 1×10^9^) and AAV-GFP-shPPP2R5C (titer: 1×10^12^) was performed stereotactically at coordinates AP +2.5 mm and ML ±2.0 mm relative to bregma, and DV -1.7 mm from the dural surface. A volume of 400 nL of viral suspension was injected into each point using a 1 μL Hamilton syringe with a fixed needle at a 100 nL/min rate. The needle remained in place for 5 min before it was removed slowly (over 2 min). The mice were placed on a heating pad until they recovered from the surgery.

#### Morris water maze test

Virus-infected Tau P301S mice underwent spatial learning and memory assessment using the Morris Water Maze (MWM), with the setup incorporating distinct extra-maze cues, as delineated in previous work.^59^ Each mouse was subjected to four trials daily for four consecutive days. Trials were spaced with a 15-min intertrial interval to minimize stress and fatigue. Subjects that failed to locate the submerged platform within 60 s were gently directed to it. Upon reaching the platform, a 15-s period was allotted to reinforce spatial learning. A probe trial was conducted on the fifth day to evaluate memory retention. The platform was discreetly removed, and the time spent in the target quadrant was recorded for 60 s, providing an index of spatial memory. Performance metrics, including latency to the platform and swim speed, were quantitatively assessed using ANY-Maze tracking software (San Diego Instruments).

#### Y maze test

The Y maze test was carried out as described previously.^60^ Each arm measured 40 cm in length, with wall heights of 12 cm, bottom widths of 3 cm, and top widths of 10 cm, converging into a central area forming an equilateral triangle with a maximum length of 4 cm. The arms were randomly designated as follows: the ‘start arm’ from which the mouse began exploring, the ‘novel arm’ initially blocked but subsequently opened for the second trial, and a third arm. During the first 5-min trial, mice had access only to the start arm and one other arm, while the novel arm remained blocked. All entries were manually recorded. After a 2-h inter-trial interval, the retention trial was initiated, granting the mice access to all three arms for 5 min to evaluate their spatial recognition memory. Mice were reintroduced into the same start arm as the first trial. The entire session was digitally recorded for subsequent analysis. The ANY-Maze software was utilized to assess the number and duration of entries into each arm. Data was interpreted by an investigator blinded to the experimental groups to ensure objectivity.

#### Golgi staining

Under deep anesthesia, the brains of mice were rapidly excised to minimize postmortem changes. The tissues were then immediately processed for a rapid Golgi staining Kit (FD Neuro Technologies, Inc., PK401), following the manufacturer’s instructions. Brain samples were submerged in the silver impregnation solution provided in the kit and stored undisturbed for two weeks in a dark environment to prevent light-induced artifacts. Following impregnation, tissues were transferred to a cryoprotectant solution for 72 h at 4°C, maintaining dark conditions to ensure optimal staining quality. Treated brains were sectioned coronally at 100-μm thickness. The sections were then carefully mounted on gelatin-coated slides to preserve structural integrity. Subsequent development of the stained sections highlighted neuronal morphology, and a clearing step was performed to enhance the contrast and visibility of dendritic structures. Dendritic spine density was quantified from secondary dendrites, selecting segments ranging from 50 to 100 μm in length from each neuron imaged. Only areas with clear visibility and evaluable morphology were included in the analysis to ensure accuracy.

#### Phosphatase assays

Total protein concentration in HEK293 cell lysates with indicated treatments was measured using the Pierce BCA protein assay kit (Thermo Fisher Scientific, #23227). Prepare six samples for each group using the following protocol. Protein lysates (100 μg) were mixed with protein A agarose slurry (30 μL) (Santa Cruz Biotechnology, #sc-2001) and 2 μL of anti-PP2A C subunit antibody (Sigma, #05–421) in a total of 500 μL with pNPP Ser/Thr assay buffer (Sigma, #20–179), and incubated for 2 h at 4°C with constant rotation. Next, the beads were washed three times with TBS, followed by one wash with 500 μL Ser/Thr assay buffer. After washing the beads, six different concentrations (0, 100, 200, 300, 400, and 500 μm) of threonine phosphopeptide (Sigma, #12–219) were added to the corresponding beads with pNPP Ser/Thr assay solution as a substrate for the enzymatic reaction. The mixture was then incubated for 10 min at 30°C in a shaking incubator. In a Corning 96-well half-area microplate (Merck, #CLS3695-25EA), combine 25 μL of the enzymatic reaction with 100 μL of Malachite Green Phosphate Detection Solution (solution A and additive, Sigma, #20–105 and 20–104, respectively) and incubate for 10 min at room temperature. Absorbance was measured at 650 nm using a SpectraMax i3x plate reader (Molecular Devices) and compared to the Phosphate standard (Sigma, #20–103).

#### Molecular docking

Molecular docking studies were performed using Rosetta 3.1 software^61^ to investigate the interactions between various ligands and proteins. In the docking procedure, the protein ULK1 (https://alphafold.ebi.ac.uk/entry/O75385) was designed as the “receptor,” while the protein PPP2R5C (ID 2IAE, Protein Data Bank)^62^ was considered as the “ligand.” The docking process commenced with a flexible blind docking approach where all heavy atoms of the protein were constrained in position. Initially, the proteins were placed randomly in proximity to each other, and the ligand-protein PPP2R5C was positioned randomly within approximately 10 Å of the receptor protein ULK1. Subsequently, 300 docking conformations were generated for each system, clustered, and ranked based on the docking energy. The top-ranked conformation exhibiting the lowest binding energy within the most prominent cluster was selected as the optimal binding mode for subsequent molecular dynamics simulations.

#### Molecular dynamics simulation

To investigate the binding modes of the docking complex, molecular dynamics (MD) simulations were performed using AMBER software (version 16)^63^ and the AMBER ff99sb force field for the complex. Hydrogen atoms were added to each starting complex model using the leap module, while ionizable residues were returned to their default protonation states at neutral pH. The complexes were solvated in a cubic periodic box of TIP3P water, extending at least 10 Å from the solute. The particle mesh Ewald (PME) approach was used with a limit of 10.0 Å from long-range electrostatic interactions.^64^ Bond lengths were restricted using the SHAKE algorithm, and a time step of 2 fs was chosen using the Varlet leapfrog approach.^65^ The system underwent a two-step minimization procedure to avoid potential confrontations between the solute and solvent. Initially, the complex was constrained by a harmonic potential (kΔxˆ2) and a force constant (k = 100 kcal/mol^−1^Å-2). Water molecules and counterions were optimized in 2500 stages using the steepest descent approach, followed by 2500 steps with the conjugate gradient method. The complete system was then minimized without constraints using the same method. After two minimization phases, an annealing simulation was run with a weak restriction (k = 100 kcal/mol^−1^Å-2), gradually heating the system from 0 to 298 K over 500 ps in the NVT ensemble. After the heating phase, MD simulations lasting more than 50 ns were performed for each system at atmospheric pressure. The Langevin thermostat with a collision frequency of 2 ps-1 was used to keep the temperature at 298 K, and the NPT ensemble employed an isotropic position scaling algorithm with a relaxation time of 2 ps to keep the pressure constant. Root-mean-square deviation (RMSD) measurements were calculated to track each complex’s conformational fluctuations. Based on the end trajectory of >100 ns MD simulations, over 1000 images were selected from the last 30 ns trajectory to determine the complex’s average structure.

### Quantification and statistical analysis

Receiver operating characteristic (ROC) curve analysis was performed to assess the diagnostic performance of plasma PPP2R5C levels in distinguishing between AD, aMCI, and CN controls, as well as between AD, PSP, and FTD. ROC curves were generated by plotting the true positive rate (sensitivity) against the false positive rate (1-specificity) at various threshold settings. The area under the curve (AUC) was calculated to evaluate the overall accuracy of PPP2R5C as a diagnostic marker. The statistical significance of the AUC was determined, and comparisons between ROC curves were conducted using DeLong’s test to assess differences in diagnostic performance among the groups.

Statistical analysis was performed with Prism 9.0 (GraphPad). All data were expressed as mean ± SEM from three or more independent experiments. Histological data were analyzed using either Student’s *t* test (two-group comparison) or one-way ANOVA with Tukey’s multiple-comparisons test. The threshold for significance for all experiments was set ∗*p* < 0.05, and smaller *p* values are represented as ∗∗*p* < 0.01 and ∗∗∗*p* < 0.001.

## Supplemental information

Document S1. Figures S1–S8 and Tables S1–S3

Document S2. Article plus supplemental information
