# Supplementary material for: Neuronal PPP2R5C in plasma is a potential biomarker for early diagnosis of Alzheimer’s disease
Source: Cell Rep Med. 2026 Feb 19;7(3):102631. doi: 10.1016/j.xcrm.2026.102631 (PMC13006427; doi:10.1016/j.xcrm.2026.102631)
Supplement: Document S1. Figures S1–S8 and Tables S1–S3 [file mmc1.pdf]

Cell Reports Medicine, Volume *7*

Supplemental information

Neuronal PPP2R5C in plasma is a potential biomarker for early diagnosis of Alzheimer's disease

Shilin Luo, Hui Liu, Tingting Xiao, Yunni Li, Xixi Liu, Xuewen Xiao, Xinxin Liao, Yingzi Liu, Yafang Zhou, Jun-Ling Wang, Jifeng Guo, Tian Tu, Xiaoxin Yan, Beisha Tang, Zhentao Zhang, Bin Jiao, and Lu Shen

Supplementary information

Neuronal PPP2R5C in plasma is a potential biomarker for early diagnosis of Alzheimer's disease

Shilin Luo, Hui Liu, Tingting Xiao, Yunni Li, Xixi Liu, Xuewen Xiao, Xinxin Liao, Yingzi Liu, Yafang Zhou, Jun-Ling Wang, Jifeng Guo, Tian Tu, Xiaoxin Yan, Beisha Tang, Zhentao Zhang, Bin Jiao, Lu Shen

Figure S1

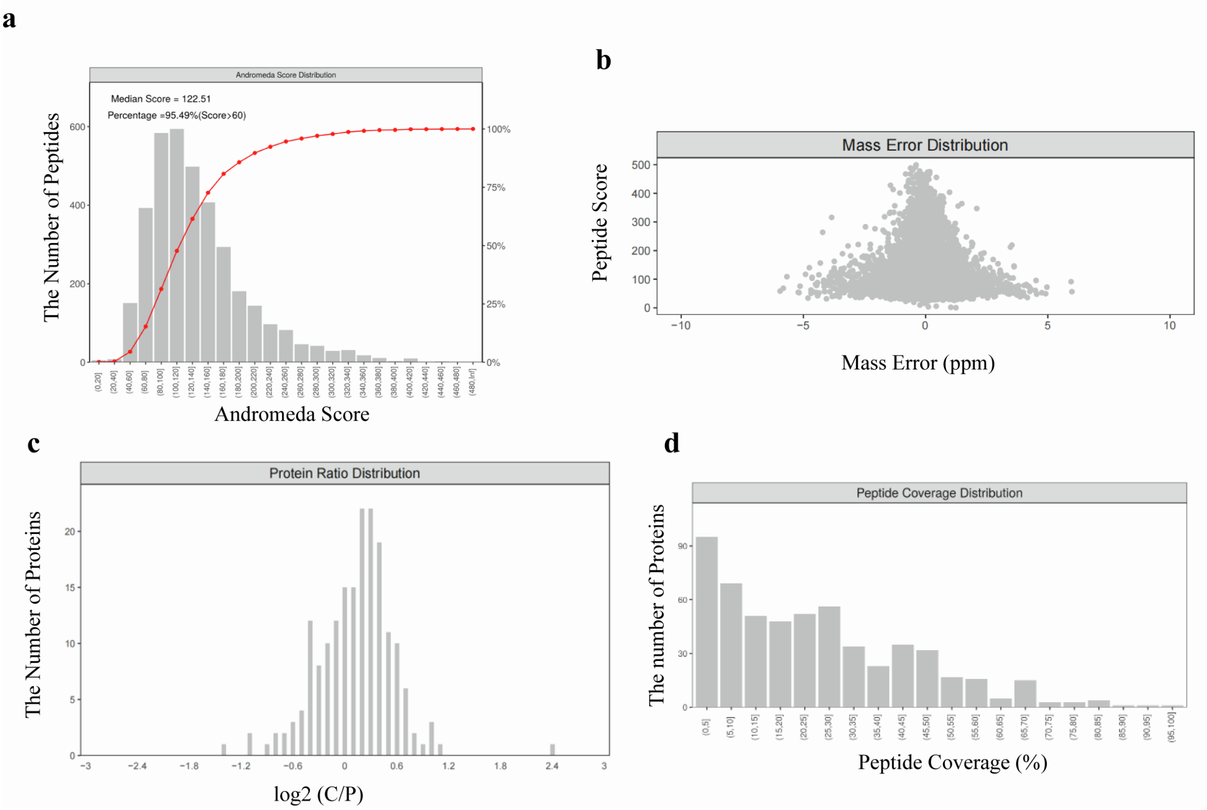

Figure S1. Quantify control of NDEs proteomics. Related to Figure 1. (a) Peptide ion score distribution map. (b) Mass error distribution map. (c) The distribution of protein expression ratios in the NDEs group analysis. (d) The protein coverage distribution data.

Figure S2

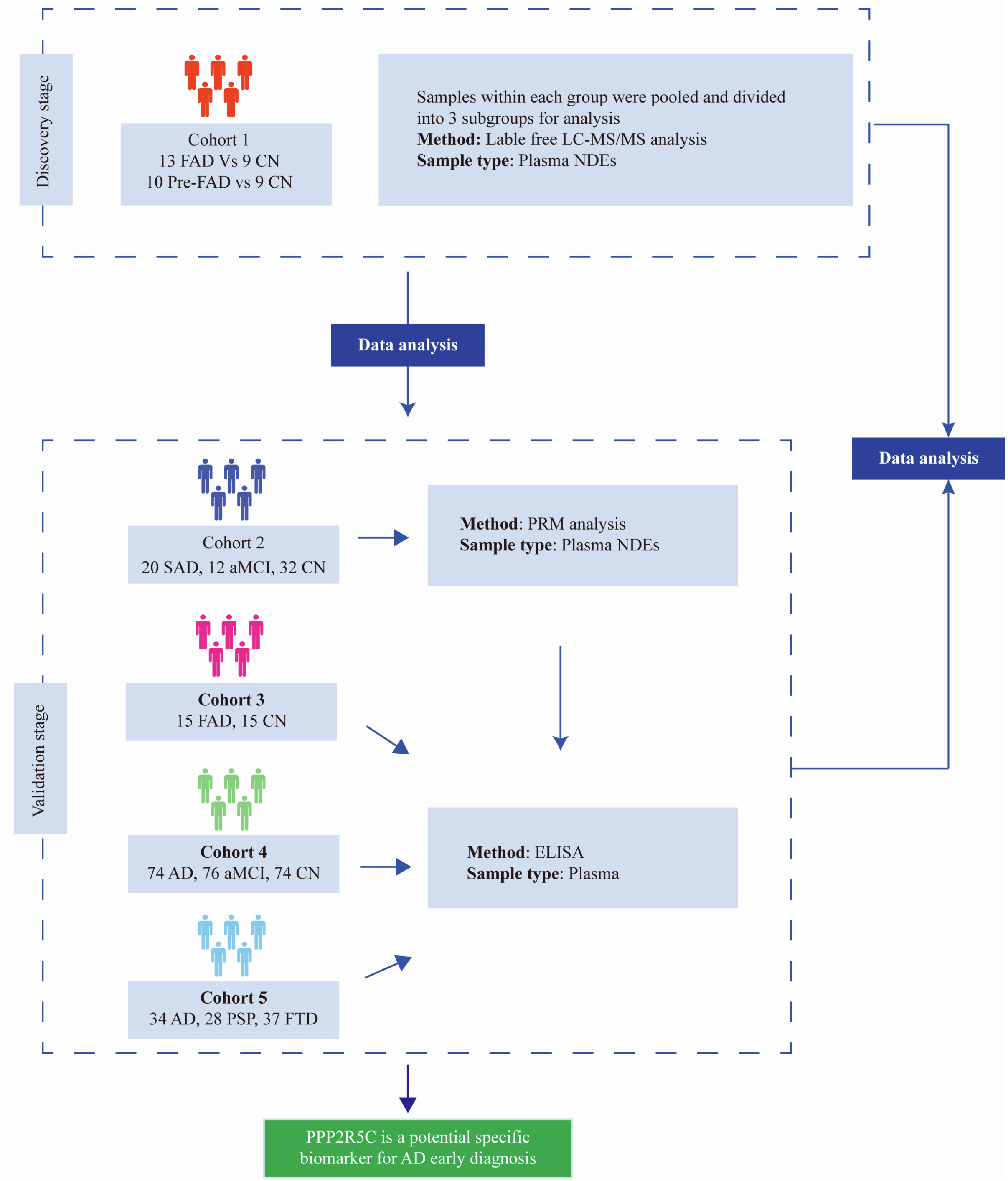

Figure S2. Flowchart for the discovery and validation process of PPP2R5C as a potential specific biomarker for AD early diagnosis. Related to Figures 1 and 2.

Figure S3

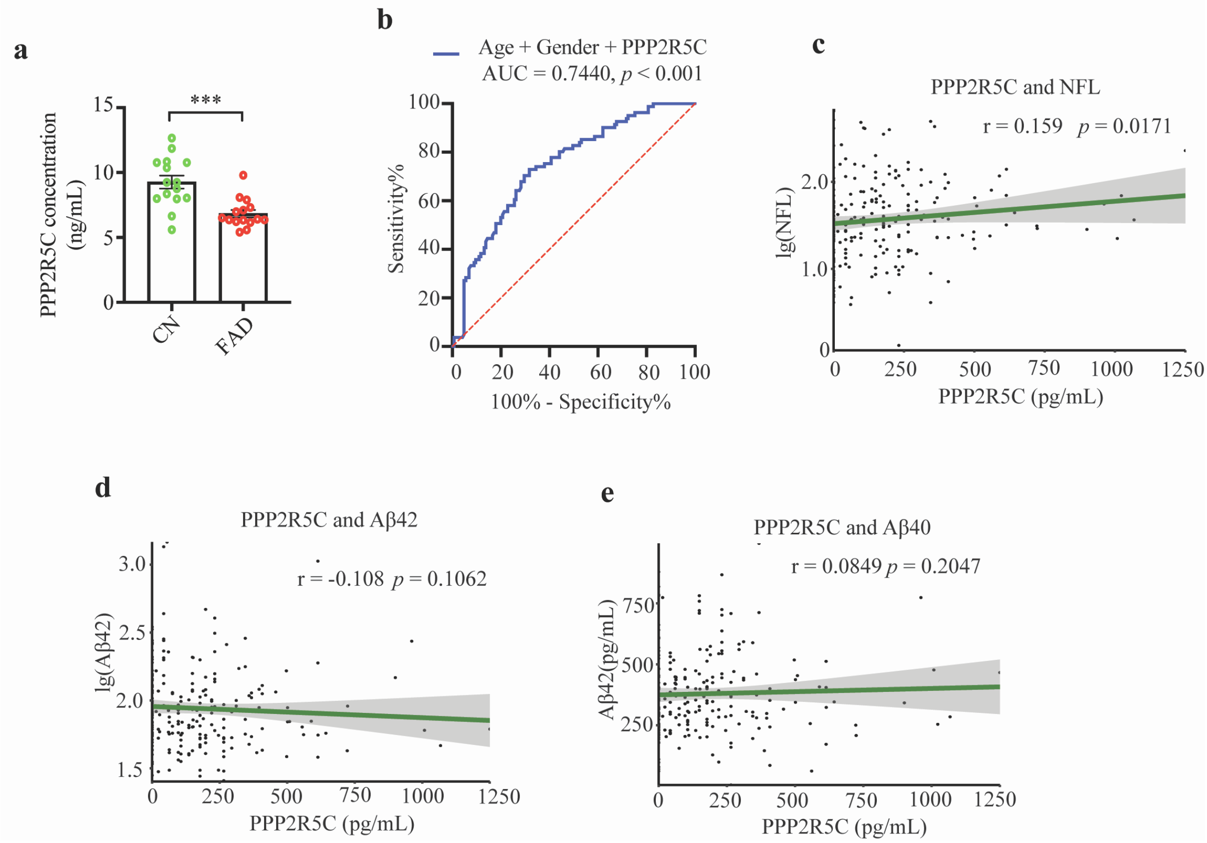

Figure S3. Plasma PPP2R5C demonstrated no significant correlations with NFL, Aβ42, and Aβ40. Related to Figure 2. (a) Plasma PPP2R5C protein levels in FAD and CN groups by ELISA technology. FAD, n = 15; CN, n = 15. (b) Evaluation of the diagnostic performance of combining PPP2R5C levels with age and gender. The correlation coefficient between plasma PPP2R5C and NFL is r = 0.159, p = 0.0171 (c); between plasma PPP2R5C and Aβ42 is r = -0.108, p = 0.1062 (d); between plasma PPP2R5C and Aβ40 is r = -0.0849, p = 0.2047 (e).

Figure S4

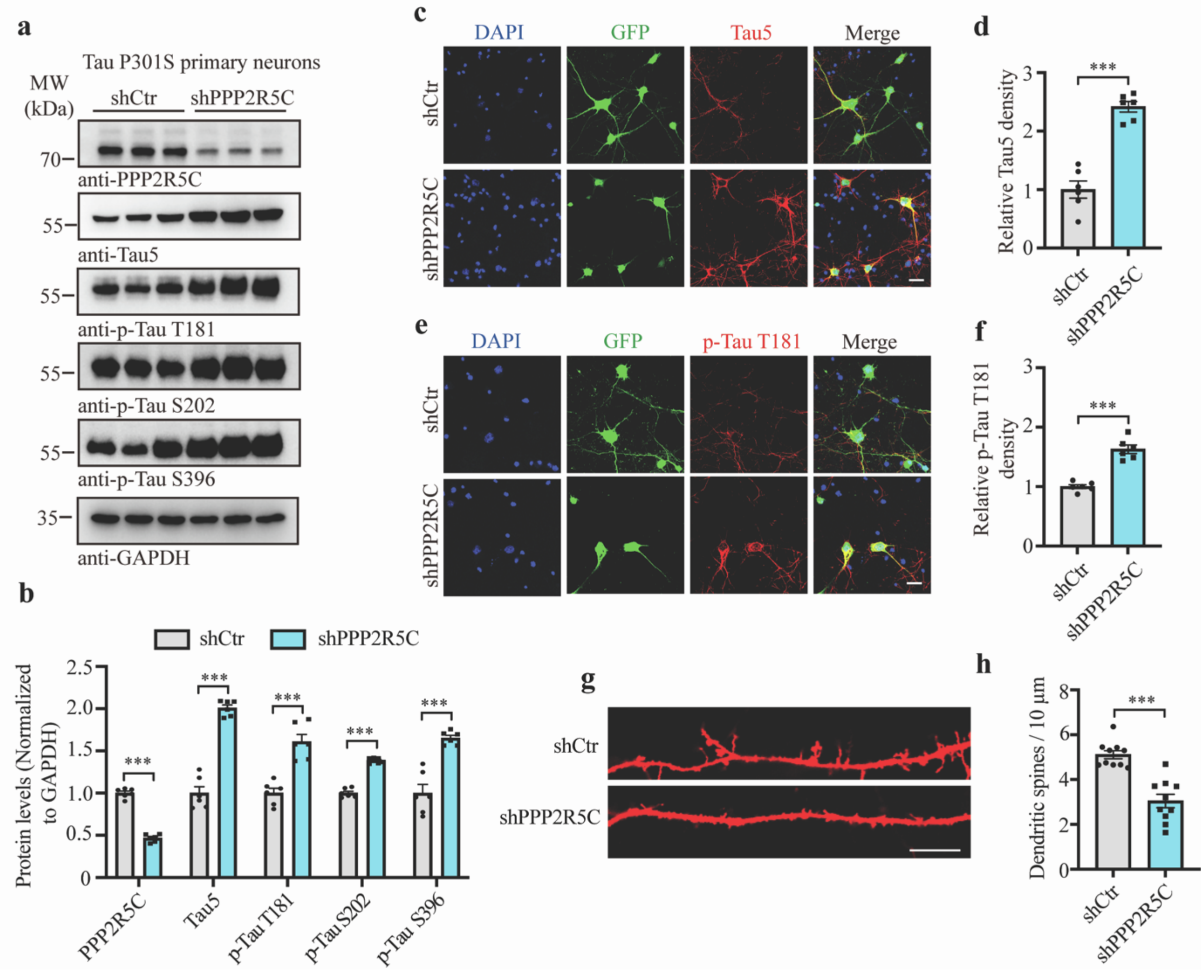

Figure S4. PPP2R5C knockdown promotes Tau and its phosphorylation levels. Related to Figure 3. (a) Representative Western blots showing t-tau, p-tau T181, p-tau S202, and p-tau S396 in primary neurons from Tau P301S mice following PPP2R5C knockdown. (b) Quantification of t-tau, p-tau T181, p-tau S202, and p-tau S396 levels. Values are normalized to sh-Ctrl. (c, e) Double-labeling immunofluorescence analysis of GFP (green) and t-tau/p-tau T181(red) was conducted on primary tau P301S neurons after PPP2R5C knockdown. Scale bar: 10 µm. (d, f) Quantification of t-tau and p-tau T181 fluorescence intensity. (g) Representative DiI staining shows the difference in spine density in Tau P301S neurons after PPP2R5C knockdown. Scale bar: 10 µm. (h) Quantification of the density of spines (n = 10). All the western blot data are representative of three independent experiments. Quantification data are expressed as mean ± SEM (*** $p < 0.001$ with Student's *t*-test).

Figure S5

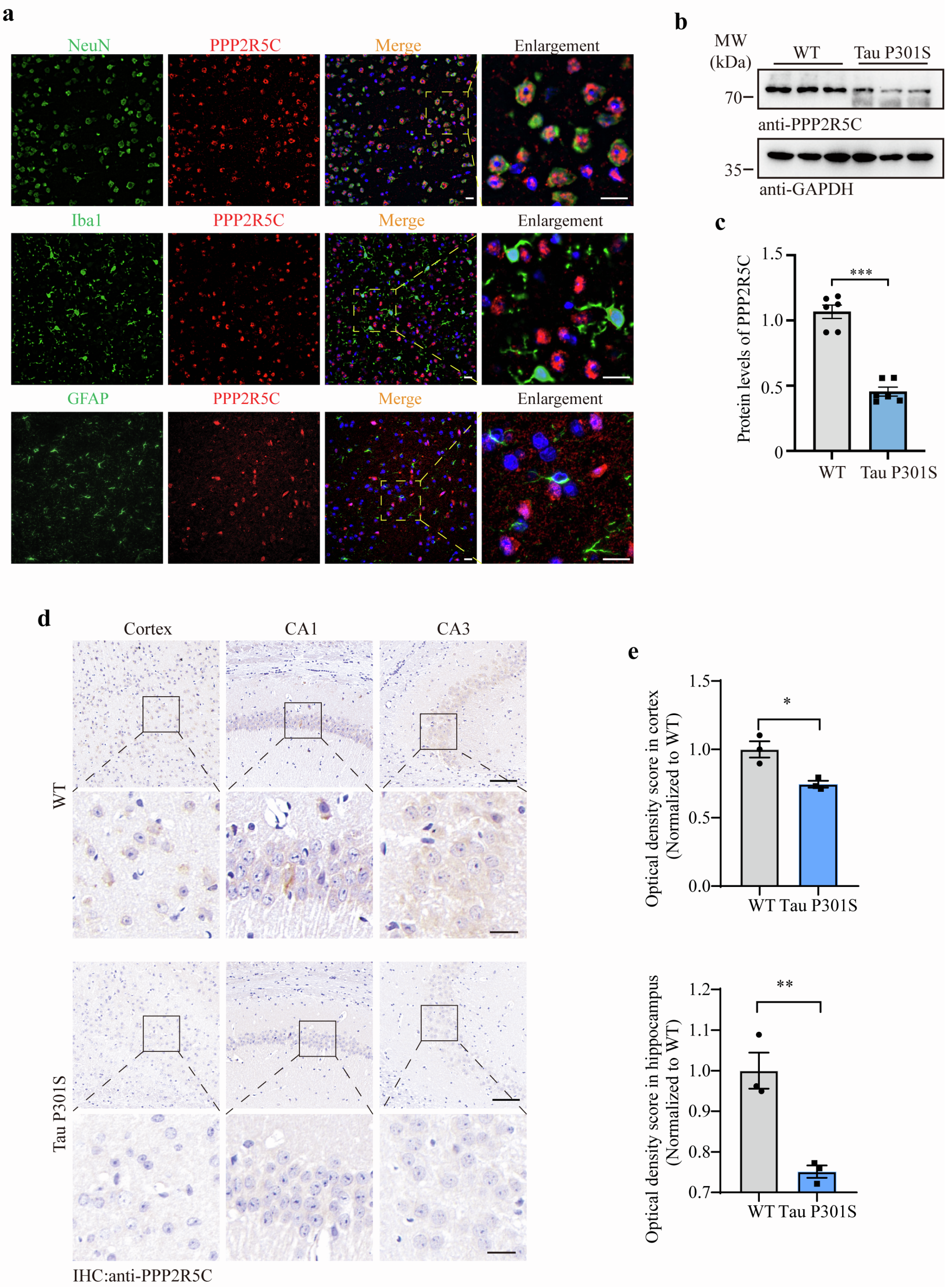

Figure S5. The expression profile of PPP2R5C in mouse brains. Related to Figure 4. (a) Double-labeling immunofluorescence analysis of PPP2R5C (red) and various cell-type markers (Green) in WT brain mouse tissues; markers include NeuN (neurons), Iba1 (microglia),

and GFAP (astrocytes). Scale bar: 10 μm (normal), 20 μm (Enlargement). (b) Western blot shows that PPP2R5C levels in the cortex of Tau P301 mice are lower than those of their WT littermates. (c) Quantification of PPP2R5C expression levels (n = 6). (d) Representative immunohistochemistry images comparing PPP2R5C protein levels in brain tissue of 6-month-old Tau P301S mice to their WT littermates. Scale bar: 100 μm (normal), 20 μm (Enlargement). (e) Quantification of IHC staining of PPP2R5C (n = 4 mice per group). The western blot data are representative of three independent experiments. Quantification data are expressed as mean ± SEM (*** $p < 0.001$ with Student's *t*-test).

Figure S6

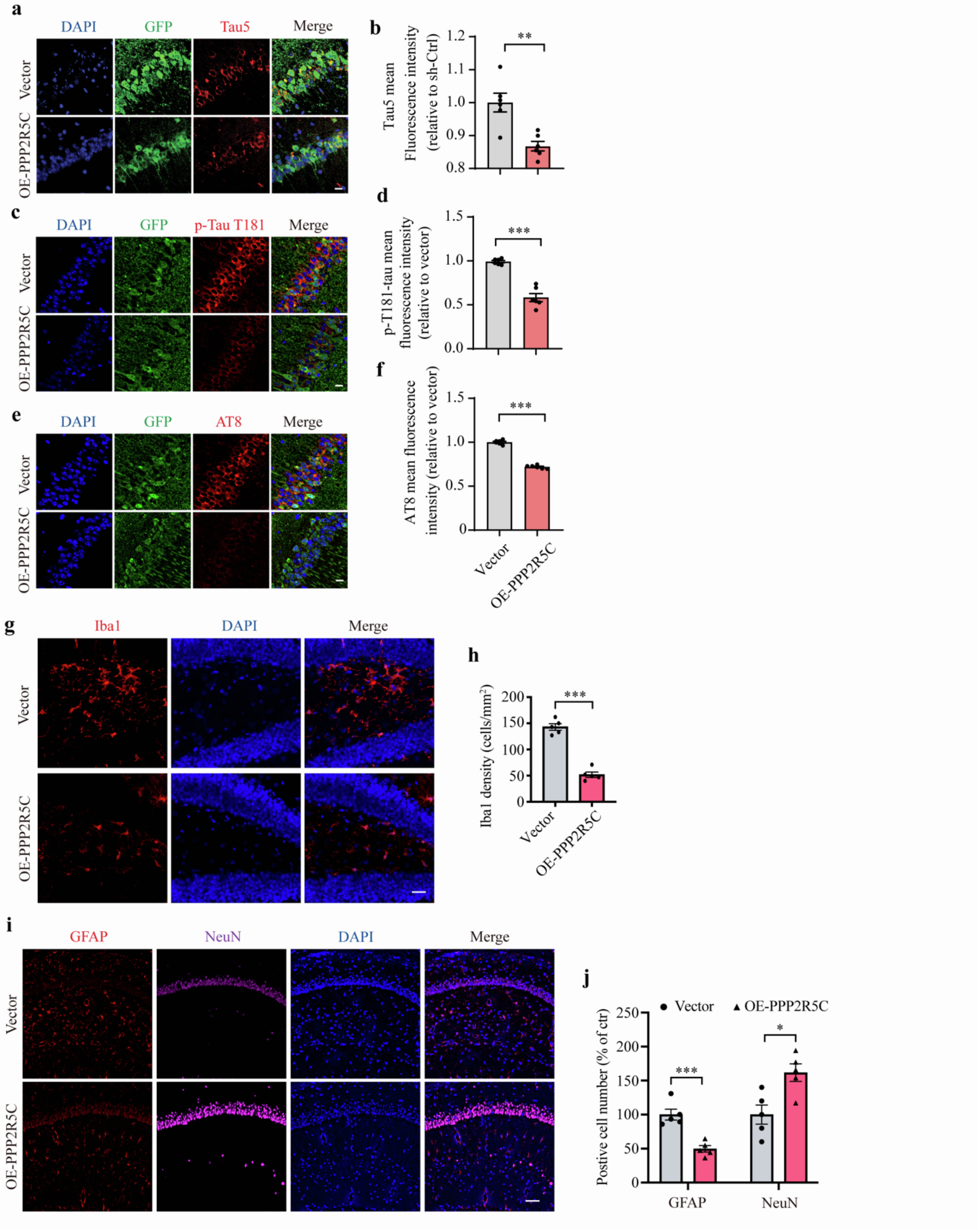

Figure S6. Overexpression of PPP2R5C decreases Tau and p-Tau levels in Tau P301S mice brains. Related to Figure 4. (a, c, e) Representative IF images of Tau (a), p-Tau T181 (c), and AT8 (e) levels on the hippocampus of Tau P301S mice after AAV-PPP2R5C/GFP or AAV-GFP infection. Scale bar: 10 µm. (b, d, f) Quantification of Tau (b), p-Tau T181 (d), and AT8 (f) fluorescence intensity (n = 6 per group). (g and i) Representative IF images of Iba1 (g),

GFAP, and NeuN (i) levels on the hippocampus of Tau P301S mice after AAV-PPP2R5C/GFP or AAV-GFP infection. Scale bar: 20 µm. (h and J) Quantification of Iba1 (g), GFAP, and NeuN (i) fluorescence intensity (n = 5 per group). Quantification data are expressed as mean ± SEM (*$p < 0.05$, **$p < 0.01$, and *** $p < 0.001$ with Student's *t*-test).

Figure S7

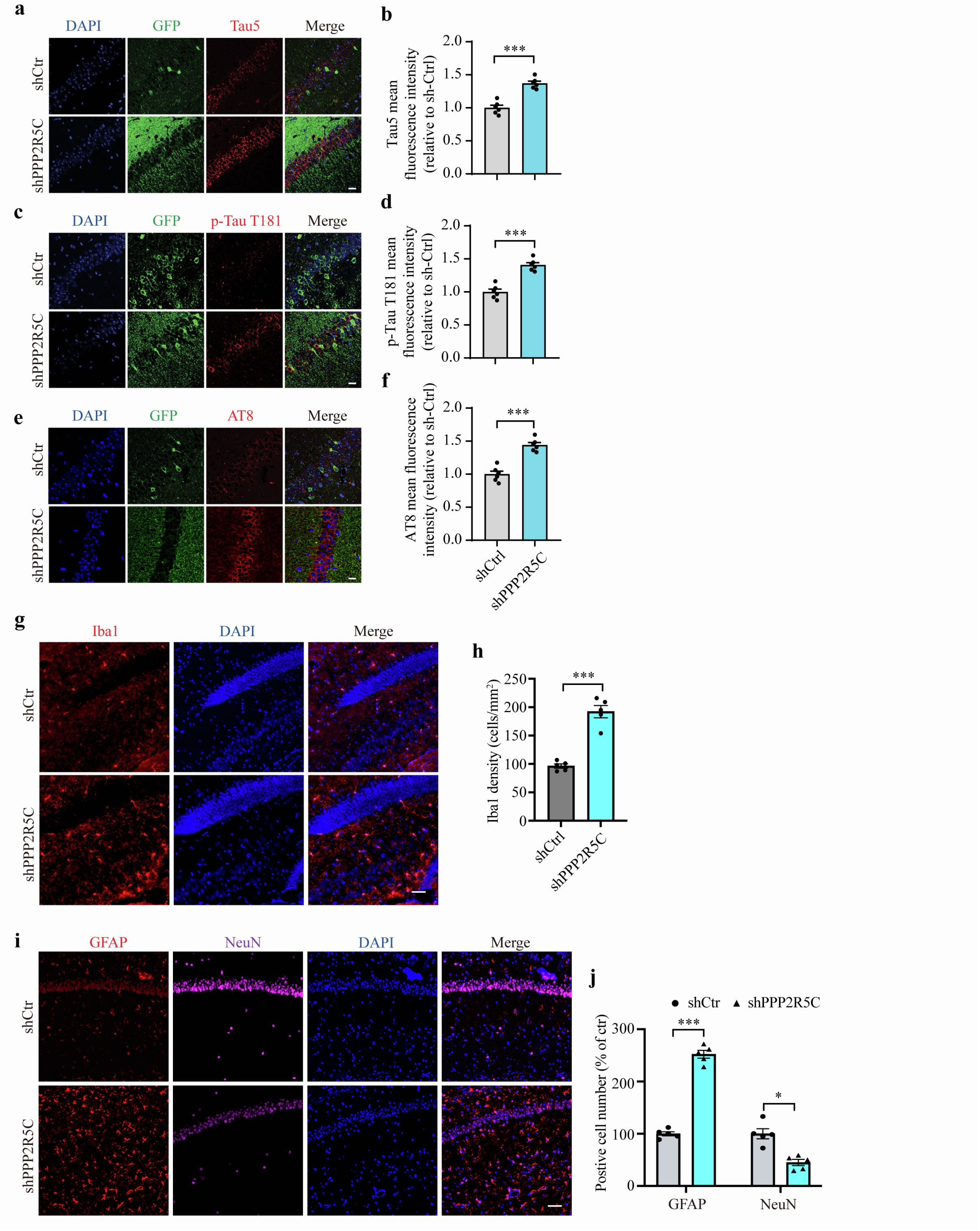

Figure S7. Blocking of PPP2R5C increased Tau and p-Tau levels in Tau P301S mice brains. Related to Figure 5. (a, c, e) Representative IF images of Tau (a), p-Tau T181 (c), and AT8 (e) levels on the hippocampus of Tau P301S mice after AAV-shPPP2R5C/GFP or AAV-shCtr infection. Scale bar: 10 µm. (b, d, f) Quantification of Tau (b), p-Tau T181 (d), and AT8 (f) fluorescence intensity (n = 6 per group). (g and i) Representative IF images of Iba1 (g),

GFAP, and NeuN (i) levels on the hippocampus of Tau P301S mice after AAV-PPP2R5C/GFP or AAV-GFP infection. Scale bar: 20 μm. (h and J) Quantification of Iba1 (g), GFAP, and NeuN (i) fluorescence intensity (n = 5 per group). Quantification data are expressed as mean ± SEM (*$p < 0.05$ and *** $p < 0.001$ with Student's *t*-test).

Figure S8

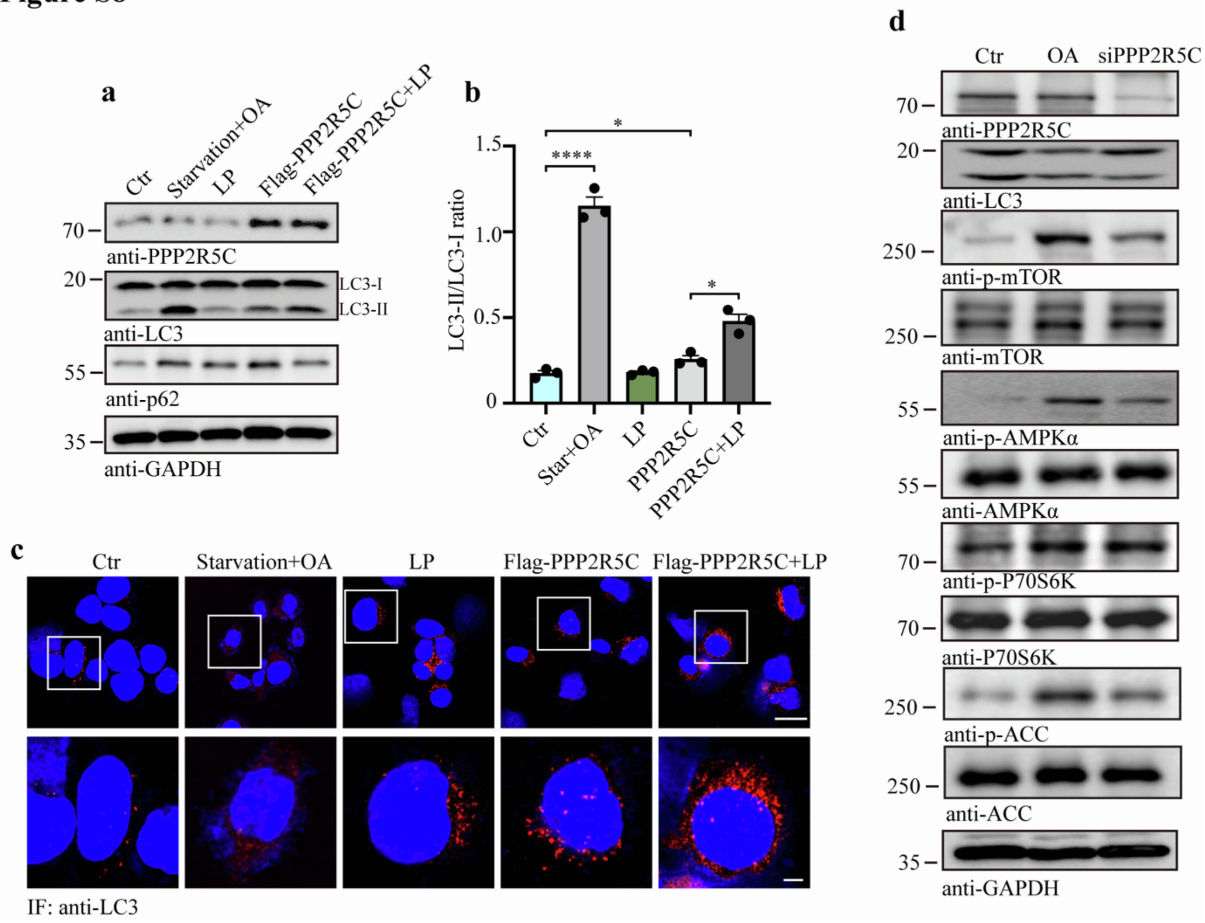

Figure S8. PPP2R5C initiates autophagy flux and affects PP2A-mediated signaling pathways. Related to Figure 6. (a) Western blot detection of the expression of LC3 and p62 in SH-SY5Y cells. PPP2R5C was transiently overexpressed in SH-SY5Y cells, and autophagy was assessed in the presence or absence of inhibitors of the autophagic flux, leupeptin, and pepstatin (LP). (b) Quantification of the ratio of LC3-II/LC3-I (n = 3). (c) Detection of autophagic vacuoles (Avs) by LC3 immunofluorescence (red). Scale bar: 10 μm (normal), 2 μm (Enlargement). (d) Western blot detecting the native and phosphorylated forms of mTOR, AMPKα, and their respective substrates P70S6K and acetyl-CoA carboxylase (ACC). Western blot data are representative of three independent experiments. Quantification data are expressed as mean ± SEM (*$p < 0.05$ and **** $p < 0.0001$ with Student's *t*-test).

Table S1 Clinical and demographic features of diagnostic cohorts

**Cohort 1**				
	CN (n = 9)	FAD (n = 13)	Pre-CN (n = 9)	Pre-FAD (n = 10)
Sex, female/male	4/5	6/7	4/5	4/6
Age, mean±SD (years)	65.53 ± 5.14	59.81.53 ± 5.14	32.50 ± 4.00	35.50 ± 5.00
MMSE	27.51 ± 0.30	11.00 ± 5.50	29.50 ± 0.25	29.07 ± 0.50
Education level, years	9.93 ± 5.04	10.60 ± 3.18	13.00 ± 2.58	15.40 ± 2.76
**Cohort 2**				
	CN (n = 32)	aMCI (n = 12)	SAD (n = 20)	
Sex, female/male	20/12	9/3	14/6	
Age, mean±SD (years)	65.59 ± 5.12	60.33 ± 9.70	62.75 ± 9.41	
MMSE, mean±SD	27.72 ± 1.75	27.00 ± 2.23	21.40 ± 2.23	
Education level, years	7.62 ± 3.58	7.75 ± 2.37	8.95 ± 3.34	
**Cohort 3**				
	CN (n = 15)		FAD (n = 15)	
Sex, female/male	8/7		8/7	
Age, mean±SD (years)	65.87 ± 6.35		67.40 ± 6.00	
MMSE, mean±SD	28.13 ± 3.00		14.21 ± 7.62	
Education level, years	12.47 ± 4.44		10.27 ± 3.08	
**Cohort 4**				
	CN (n = 74)	aMCI (n = 76)	AD (n = 74)	
Sex, female/male	43/31	35/41	38/36	
Age, mean±SD (years)	67.03 ± 6.09	65.91 ± 9.27	60.40 ± 8.47	
Education level, years	7.23 ± 1.31	9.61 ± 4.52	8.86 ± 3.69	
MMSE	29.39 ± 0.48	25.87 ± 2.81	15.24 ± 7.17	
PPP2R5C, mean±SD (pg/mL)	256.5 ± 239.6	157.2 ± 227.1	81.80 ± 107.4	
p-tau T181, mean±SD (pg/mL)	4.23 ± 1.23	11.62 ± 9.92	43.12 ± 86.99	
p-tau T217, mean±SD (pg/mL)	3.58 ± 0.85	5.32 ± 2.71	14.47 ± 7.25	
p-tau S231, mean±SD (pg/mL)	7.60 ± 3.18	20.42 ± 21.90	501.0 ± 671.3	
**Cohort 5**				
	AD (n = 34)	PSP (n = 28)	FTD (n = 37)	
Sex, female/male	19/13	11/27	25/31	
Age, mean±SD (years)	57.47 ± 9.53	63.47 ± 7.00	62.11 ± 8.94	
Education level, mean±SD (years)	10.07 ± 4.16	7.94 ± 3.63	7.56 ± 3.58	
MMSE	15.38 ± 7.36	21.31 ± 7.77	14.69 ± 8.59	
PPP2R5C, mean±SD (pg/mL)	160.3 ± 96.23	430.6 ± 320.1	278.8 ± 265.5	

Table S2 The information on the diagnostic efficacy of PPP2R5C combining p-tau217 and other conventional variables, related to Figure 2.

	Groups	AUC	specificity	sensitivity	accuracy
PPP2R5C	CN-MCI	0.736	0.506494	0.932432	0.715232
	CN-AD	0.8494	0.64	0.972973	0.805369
	MCI-AD	0.5931	0.64	0.584416	0.611842
p-tau217	CN-MCI	0.7898	0.675325	0.810811	0.741722
	CN-AD	0.998	0.973333	0.986487	0.979866
	MCI-AD	0.9468	0.96	0.831169	0.894737
PPP2R5C-p-tau217	CN-MCI	0.8475	0.766234	0.837838	0.801325
	CN-AD	0.9991	0.986667	1	0.993289
	MCI-AD	0.9533	0.946667	0.883117	0.914474
p-tau217-age-sex	CN-MCI	0.7893	0.605263	0.878378	0.74
	CN-AD	0.9993	0.986667	0.986487	0.986577
	MCI-AD	0.9475	0.906667	0.881579	0.89404
p-tau217-age-sex-PPP2R5C	CN-MCI	0.8321	0.736842	0.810811	0.773333
	CN-AD	0.9995	1	0.986487	0.993289
	MCI-AD	0.9544	0.946667	0.842105	0.89404
p-tau217-age-sex-decation years	CN-MCI	0.8373	0.81579	0.810811	0.813333
	CN-AD	0.9991	0.973333	1	0.986577
	MCI-AD	0.9465	0.906667	0.868421	0.887417
p-tau217-age-sex-decation years-PPP2R5C	CN-MCI	0.8578	0.763158	0.905405	0.833333
	CN-AD	1	1	1	1
	MCI-AD	0.9568	0.973333	0.868421	0.92053

Table S3 Information of human post-mortem brain samples, related to Figure 2.

	No.	Case ID	Age	Gender	Post mortem delay (h)	Braak
Health Ctr	1	XYA20240829	82	Male	5	0
	2	XYA20220609	65	Male	3	0
	3	XYA20190916	76	Male	7	0
	4	XYA20221204	71	Male	5	I
	5	XYA20240902	60	Male	7	0
AD1	1	XYA20241012	77	Male	5.5	II
	2	XYA20210207	69	Male	6	II
	3	XYA20230423	76	Male	5	II
	4	XYA20240628	72	Male	10	II
	5	XYA20230117	93	Male	8	II
	6	XYA20231103	87	Female	8	II
	7	XYA20240208	72	Male	9	II
	8	XYA20210926	68	Female	7	II
AD2	1	XYA20240717	75	Male	8	III
	2	XYA20230111A	88	Male	6	III
	3	XYA20221228B	95	Male	6	III
	4	XYA20220406	83	Female	11	III
	5	XYA20201014	90	Male	9	III
AD3	1	XYA20240926	94	Male	7	IV
	2	XYA20240714	96	Female	10	IV
	3	XYA20230106A	69	Male	10	IV
	4	XYA20220727	88	Female	6	IV
	5	XYA20220810	86	Male	3	IV
